# Emerging roles of lactate in acute and chronic inflammation

**DOI:** 10.1186/s12964-024-01624-8

**Published:** 2024-05-16

**Authors:** Yunda Fang, Zhengjun Li, Lili Yang, Wen Li, Yutong Wang, Ziyang Kong, Jia Miao, Yanqi Chen, Yaoyao Bian, Li Zeng

**Affiliations:** 1 School of First Clinical Medicine, Nanjing University of Chinese Medicine, Nanjing, 210023 China; 2 Jiangsu Provincial Engineering Research Center of TCM External Medication Development and Application, Nanjing University of Chinese Medicine, Nanjing, 210023 China; 3 College of Health Economics Management, Nanjing University of Chinese Medicine, Nanjing, 210023 China; 4 Jingwen Library, Nanjing University of Chinese Medicine, Nanjing, 210023 China; 5 School of Acupuncture-Moxibustion and Tuina, ·School of Health Preservation and Rehabilitation, Nanjing University of Chinese Medicine, Nanjing, 210023 China; 6 TCM Rehabilitation Center, Jiangsu Second Chinese Medicine Hospital, Nanjing, 210023 China; 7 Faculty of Chinese Medicine, Macau University of Science and Technology, Taipa, Macau, 999078 China

**Keywords:** Inflammatory response, Lactate metabolism, Lactylation, Lactate clock, Glycolysis

## Abstract

Traditionally, lactate has been considered a ‘waste product’ of cellular metabolism. Recent findings have shown that lactate is a substance that plays an indispensable role in various physiological cellular functions and contributes to energy metabolism and signal transduction during immune and inflammatory responses. The discovery of lactylation further revealed the role of lactate in regulating inflammatory processes. In this review, we comprehensively summarize the paradoxical characteristics of lactate metabolism in the inflammatory microenvironment and highlight the pivotal roles of lactate homeostasis, the lactate shuttle, and lactylation (‘lactate clock’) in acute and chronic inflammatory responses from a molecular perspective. We especially focused on lactate and lactate receptors with either proinflammatory or anti-inflammatory effects on complex molecular biological signalling pathways and investigated the dynamic changes in inflammatory immune cells in the lactate-related inflammatory microenvironment. Moreover, we reviewed progress on the use of lactate as a therapeutic target for regulating the inflammatory response, which may provide a new perspective for treating inflammation-related diseases.

Inflammation is a protective response that is triggered by exogenous or endogenous stimuli (such as infection or tissue damage) and is characterized by the activation of immune and non-immune cells, the alleviation of injury, the clearance of necrotic cells, and the promotion of tissue repair [1]. The inflammatory response is one of the primary lines of defence of the host that is used to resist pathogen invasion [2]. When cells recognize pathogen-associated molecular patterns or damage-associated molecular patterns, they rapidly produce and release proinflammatory cytokines and chemokines to trigger inflammatory responses [3]. These extracellular factors recruit immune cells to the site of injury to eliminate microbial infection and modulate the inflammatory response. Under physiological conditions, the inflammatory response will dissipate gradually and automatically as the threat is eliminated [4]. However, if persistent inflammation hinders the dissipation of the inflammatory response and activates inflammasomes by recognizing damage-associated molecular patterns, the body will experience chronic inflammation [5]. Continuous low-grade tissue infiltration of immune cells activates the inflammasome, impairs normal immune function, and amplifies the inflammatory response, which may lead to severe tissue damage and remodelling, thereby inducing the development of chronic diseases [6]. Inflammation involves multiple organs in the body, and damage to these organs may have severe clinical consequences. According to the Global Burden of Disease Study, more than 50% of deaths can be attributed to inflammation-related diseases, which are recognized as one of the most common causes of death worldwide today and impose considerable psychological and economic burdens on patients and society [3, 7]. Another study reported that there were approximately 48.9 million cases of sepsis, which is generally viewed as a condition of overwhelming systemic inflammation [8], and 11.0 million sepsis-related deaths occurred in 2017, representing 19.7% of all global deaths [9]. Therefore, researchers have been trying to find anti-inflammatory therapies with low toxicity that regulate the inflammatory response, do not promote antibiotic resistance, and reduce mortality [10].

As a major source of energy required for the body, glucose plays a key role in the maintenance of biological activities [11]. Under normal aerobic conditions, glucose can be catabolized by glycolysis into two pyruvate molecules, two adenosine triphosphate (ATP) molecules and two nicotinamide adenine dinucleotide (NADH) molecules [12]. Pyruvate enters the tricarboxylic acid (TCA) cycle in the form of acetyl-CoA via pyruvate dehydrogenase (PDH) and generates up to approximately 32 ATP molecules after oxidative phosphorylation (OXPHOS) [13]. When oxygen consumption or the oxygen supply of body tissues and cells is insufficient under conditions of intense exercise or diseases such as tumours, inflammation, infection, and ischaemia, the TCA cycle is inhibited, and the aerobic glycolysis pathway is activated to compensate for the lack of ATP production by OXPHOS [14]. Moreover, pyruvate and NADH are reduced to lactate and NAD^+^ by lactate dehydrogenase (LDH), thus allowing glycolysis to persist. Previously, lactate was considered the metabolic waste product of glycolysis. However, recent research has demonstrated that glucose may play a supportive role in bioenergetic metabolism in T cells, while lactate serves as a more favourable carbon source for the TCA cycle [15–18]. As an important signalling molecule, lactate also plays a crucial role in regulating the immune response, thus affecting immune surveillance and escape-related behaviours.

Therefore, targeted regulation of lactate is a promising therapeutic approach for the management of inflammation-related diseases. The identification of lactylation, a recently discovered posttranslational protein modification, has greatly improved our understanding of the functional roles and implications of lactate in various diseases, such as tumours and inflammatory disorders [19]. Moreover, this study paves the way for novel research into the critical roles of lactate in tumour biology, inflammation, immunity, and related fields. This review summarizes the paradoxical role of lactate and lactate metabolism in the inflammatory microenvironment and highlights the essential role of lactate in the regulation of both acute and chronic inflammatory processes with the aim of providing a new perspective for the prevention and treatment of inflammatory diseases.

## Lactate and lactate metabolism in the inflammatory microenvironment

### The ‘Warburg effect’ in the inflammatory microenvironment

The ‘Warburg effect’, originally discovered by Otto Warburg in cancer, is characterized by a shift in cellular metabolism from OXPHOS to aerobic glycolysis [20]. Mounting evidence shows that the ‘Warburg effect’ occurs not only in tumors, but also in the pathogenesis of non-cancerous diseases, such as inflammatory diseases [21, 22].

Most immune cells are relatively quiescent in a stable state, but when the body is subjected to microbial infection, tissue damage, or cellular stress, immune cells can rapidly respond to activate metabolic pathways for inflammatory reactions to help the body eliminate threats and restore homeostasis [23]. The transcription factor hypoxia-inducible factor 1 α (HIF-1α) translocates to the nucleus and binds to glycolysis-regulated genes to induce the ‘Warburg effect’ and the production of lactate [24, 25].

Contrary to previous assumptions that lactate is simply metabolic waste, lactate serves as a vital carbon source for the TCA cycle in T cells and macrophages, with a preference even surpassing that of glucose. Compared with those cultured with glucose alone, in vitro-activated CD8^+^ T cells cultured with 2 mM lactate exhibited a significant 15%–20% increase in basal ATP production and 30% increase in maximal ATP production from OXPHOS [15]. LDHB converts lactate to pyruvate, which is further oxidized in the mitochondria and subsequently metabolized by the TCA cycle to citrate, malate, and α-ketoglutarate [18, 26, 27]. These metabolites generated by lactate augment the inflammatory response, notable examples being citrate which can induce NO, prostaglandins, and ROS production [28]. Therefore, lactate plays a pivotal role in inflammatory diseases and represents a potential therapeutic target for their treatment.

### Lactate acidifies the extracellular environment and affects cellular function

The glycolytic metabolic rate increases in response to the increased demand for energy in inflammatory cells, resulting in augmented production and release of lactate. Excess lactate is exported via monocarboxylate transporters (MCTs)1–4 and converted to lactic acid by a proton (H^+^) acquired from NADH. Lactic acid, a weakly hydrophilic acid, is easily converted to lactate and H^+^, which can cause acidification in the extracellular environment [29]. Acidosis caused by the accumulation of lactate, which is an end product of the glycolytic pathway, in the extracellular environment has become a hallmark of inflammatory diseases and cancer [30–34]. The physiological lactate concentration in blood and tissues is approximately 1.5–3 mM [35], the blood lactate concentration in sepsis patients can reach more than 20 mM [36], and the lactate concentration in inflammatory tissues can even reach 40 mM [33]. Lactic acid induces a decrease in the intracellular pH by entering the cytosol of cytotoxic CD8^+^ T lymphocytes, thereby inhibiting the proliferation of CD8^+^ T cells, cytokine production, and cytotoxic function. However, a recent study demonstrated that the inhibition of lactate by LDH knockdown leads to a reduction in antigen-specific T-cell expansion and interferon (IFN)-γ production. Moreover, the accumulation of lactate initiates lactylation, which transits macrophages from the M1 phenotype with high glycolytic/low TCA activity to the M2 phenotype with low glycolytic/high TCA activity, thereby promoting wound healing and the resolution of immune responses during inflammation [27]. These findings indicate that lactate and lactate metabolism can affect cellular function, amplify inflammatory responses or induce the resolution of inflammation [37, 38].

## Factors involved in lactate metabolism in the inflammatory microenvironment

### Enzymes participate in lactate production

**Fig. 1:**
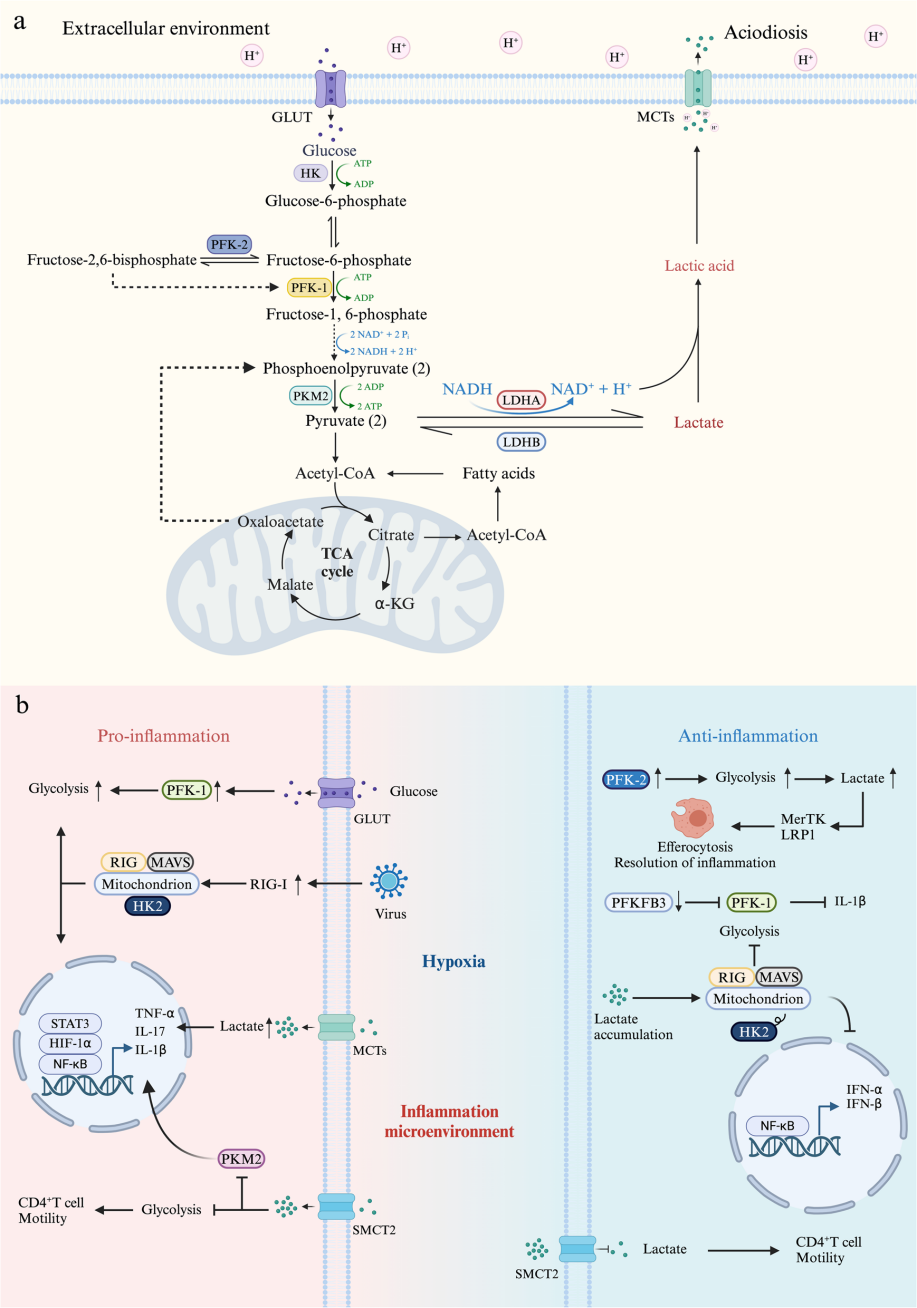
**a** Key enzymes the participate in glycolysis. In the inflammatory microenvironment, the rate of glycolysis increases to facilitate rapid energy production, resulting in an increase in lactate synthesis. On the one hand, the accumulation of lactate acidifies the extracellular space, thus impairing immune cell viability and functionality. On the other hand, lactate serves as a crucial carbon source for immune cells and acts as a vital metabolic fuel during the later stages of inflammation. **b** Lactate-related receptors in the inflammatory microenvironment. Lactate metabolism plays crucial roles during proinflammatory and anti-inflammatory processes in maintaining inflammation-environmental homeostasis. Lactate is transported by specific receptors to exert proinflammatory or anti-inflammatory effects in the inflammatory microenvironment.

To rapidly meet the high energy requirements of inflammatory cell recruitment and microbial phagocytosis, infiltrating cells in the inflammatory microenvironment are highly glycolytic. Several enzymes are involved in glucose glycolysis that leads to the production of lactate, which serves as an important carbon source for cell metabolism and enables the transition between glycolysis, OXPHOS, and fatty acid oxidation by adjusting the NAD-to-NADH ratio via CD40 signaling [39, 40]. The key enzymes that participate in the process of glycolysis are presented in Fig. 1a.

#### Hexokinase 2 (HK2)

HK is the first rate-limiting enzyme in glycolysis, and four subtypes of human HK have been identified: HK1, HK2, HK3, and HK4 [41]. Extracellular glucose enters the cell via glucose transporters and is phosphorylated by HK with the consumption of ATP to produce glucose-6-phosphate, which is the first step in glucose metabolism. Glucose-6-phosphate is converted to pyruvate by PKM2 and then to lactate by LDHA. HK plays a key role in energy metabolism, gene transcription, and other processes [42]. HK1 and HK2 bind to the mitochondrial outer membrane and preferentially contact mitochondrial ATP to promote glycolysis [43]. As a sensor for changes in cellular glucose levels, HK2 detects glucose availability through glucose-6-phosphate-mediated detection, regulates the aerobic glycolysis rate and lactate production, and ensures cell energy homeostasis and cell survival [44]. Repressed glycolysis is commonly associated with alleviated inflammation [45]. A potential therapeutic approach for inflammatory diseases involves the inhibition of HK2, thereby reducing the rate of glycolysis and subsequently mitigating the inflammatory response [46–48]. However, Hu et al discovered that deficiency of HK2 hampers microglial glycolysis and impairs mitochondrial function, thereby promoting neuroinflammation through dysfunctional mitochondria and accumulated ROS [49].

#### Phosphofructokinase-1 (PFK-1)

PFK-1, the second rate-limiting enzyme in glycolysis [50], phosphorylates fructose-6-phosphate to fructose-1,6-diphosphate, which constitutes a critical control point for regulating glycolytic flux [51]. PFK-1 is a tetramer protein, and three genes encode the human subtype: PFK-M (expressed in muscle tissue), PFK-L (expressed in the liver), and PFK-P (found in the plasma) [52]. The rate of PFK-1 in the cytoplasm is influenced by various allosteric effectors, of which fructose-2,6-bisphosphate is the most potent activator [53]. LPS enhances PFK-1 activity, leading to an increased rate of glycolysis and the production of lactate. Downregulation of PFK-1 expression by the inhibition of PFKFB3, which promotes fructose-2,6-bisphosphate production, reverses the LPS-induced inflammatory effect that promotes the secretion of IL-1β [46].

#### PFK-2

PFK-2 is composed of four genes encoding PFKFB1-4 [54]. Despite sharing a sequence homology of 85%, the four isoenzymes exhibit variations in tissue-specific expression profiles, kinase/phosphatase activity ratios, and responses to protein kinase, hormone, and growth factor signalling [55]. PFK-2 catalyses both the synthesis and degradation of fructose-2,6-bisphosphate, which is an activator of PFK-1, thereby controlling the speed of glycolysis [56]. Inhibition of PFK-2 leads to reduced NF-κB activation, thereby inhibiting the expression of adhesion molecules, proinflammatory cytokines, and chemokines [57]. Resolving-type macrophages, which mainly rely on OXPHOS, prevent chronic inflammation by clearing apoptotic cells through efferocytosis. Efferocytosis promotes a transient increase in macrophage glycolysis and lactate production, which is dependent on the rapid activation of PFKFB2. Lactate facilitates persistent efferocytosis to resolve chronic inflammation by upregulating MerTK and LRP1 expression [58]. Upregulation of PFKFB3 expression can promote glycolysis to fulfil the heightened energy and metabolic demands in inflamed tissues [59].

#### Pyruvate kinase M (PKM) 2

Pyruvate kinase is the third rate-limiting enzyme in the glycolysis pathway and has three isoenzymes, M type, R type, and L type. Notably, M type can be further classified into two subtypes, M1 and M2 [60]. PKM2 can promote glycolysis to convert phosphoenolpyruvate to pyruvate [61]. PKM2 is a pivotal regulator of various biological processes, including HIF-1α stabilization, IL-1β production, macrophage polarization, glycolytic reprogramming, ‘Warburg’ metabolism, and inflammasome activation [62, 63]. Lactate induces nuclear translocation of PKM2 in CD4^+^ T cells, leading to increased expression of phosphorylated STAT3 and interleukin (IL)-17, which drives the inflammatory response [33]. PKM2 expression is substantially increased in lipopolysaccharide (LPS)-activated macrophages and promotes the production of lactate. Then, lactate inhibits glycolysis by increasing the nuclear translocation of PKM2 via a feedback loop, thereby stimulating macrophages to shift from a proinflammatory phenotype to a reparative phenotype [64].

#### LDH

LDH is an NAD^+^-dependent enzyme that exists in two isoforms, LDHA and LDHB, and mediates the bidirectional conversion of pyruvate and lactate. LDH-5 (A4) has a greater affinity for pyruvate than for lactate and promotes the conversion of pyruvate to lactate, whereas LDH-1 (B4) has a greater affinity for lactate than pyruvate, thus favouring the conversion of lactate to pyruvate [65]. LDHA catalyses the conversion of pyruvate to lactate and NAD^+^. In an inflammatory environment, excessive glycolysis and the production of lactate may cause acidification of the cytoplasm, thereby inducing conformational changes and decreasing LDHA activity [66]. Conversely, LDHB facilitates the reduction of lactate back into pyruvate, thereby allowing lactate to serve as a source for oxidative metabolism and/or gluconeogenesis [67].

### Lactate affects immune cell function through receptors

As a main product of glycolysis, lactate is sensed by immune cells through the expression of receptors, affects immune cell metabolism, and regulates the anti-inflammatory or proinflammatory cell phenotypes [68]. Lactate-related receptors that are present in the inflammatory microenvironment are presented in Fig. 1b.

#### MCT

MCT is a member of the solute carrier 16 transporter family, of which there is a total of 14 subtypes [69]. Among them, MCT1-4 is a proton-dependent transporter involved in catalysed proton coupling and the bidirectional transport of monocarboxylic acid; this transporter exports lactate and protons to maintain a high rate of glycolysis and alkaline intracellular pH by recycling NADH [70–72]. The lactate shuttle transports lactate as a vehicle between highly glycolytic cells (producer) and highly oxidative cells (consumer). As both a product of glycolysis and a substrate for mitochondrial respiration, lactate is the link between glycolytic and oxidative metabolism [73]. Under physiological conditions, MCT1-4 promotes the shuttling of lactate between glycolytic and oxidizing cells, and the high affinity of MCT1 is responsible for the transfer of lactate according to the transmembrane lactate gradient to maintain intracellular and extracellular lactate homeostasis [74]. MCT1 is a key receptor in glycolysis that promotes the diffusion and transport of lactate, which can enhance the expression of 6-phosphofructo-2-kinase/fructose-2,6-bisphosphatases (PFKFB)3 through HIF-1α, induce the activation of inflammatory cells, and promote the inflammatory response [75]. Inflammatory cells and other cells with high intracellular lactate concentrations rely on MCTs to transfer lactate, which may contribute to the activation of transcription factor nuclear factor kappa-B (NF-κB), thereby promoting the expression of inflammatory factors and aggravating the inflammatory response [76].

#### Sodium-coupled monocarboxylate transporter (SMCT) 2

Solute carrier (SLC) 5 is a family of sodium-coupled transporter proteins; there are 12 members of SLC5A (1–12) [35, 77], among which SMCT1 (SLC5A8) and SMCT2 (SLC5A12) are SMCT transporters [78]. Despite structural and mechanistic differences from MCTs, SMCTs share several substrates with MCTs, including lactate [79]. In contrast to MCT1, the primary lactate transporter in macrophages, SMCT2 acts as a low-affinity transporter and serves as the predominant carrier for lactate entry into CD4^+^ T cells. SMCT2 facilitates the infiltration of CD4^+^ T cells into inflammatory tissues and promotes their differentiation into T helper (Th)17 cells and various subsets of T cells [80]. The inhibition of SMCT2 can reduce the uptake of lactate, restore T-cell motility, and alleviate the Th17-mediated inflammatory response [33].

#### Mitochondrial antiviral signalling proteins (MAVS)

MAVS mediates the activation of NF-κB and interferon regulatory factor 3, thereby stimulating the RIG-I-like receptor signalling pathway and promoting the production of IFN in response to viral infection [81]. As a sensor of lactate [82], MAVS binds with lactate generated by glycolysis in tissues affected by the ‘Warburg effect’, such as inflamed tissues. Upon binding with lactate, MAVS releases HK2, which does not bind to mitochondria and becomes inactivated, consequently hindering IFN generation and leading to immune evasion [83]. These findings suggest that MAVS not only plays a dual role in regulating glycolysis and innate immune responses but also participates in maintaining HK2 activity.

### Lactate signalling pathways in the inflammatory response

**Fig. 2:**
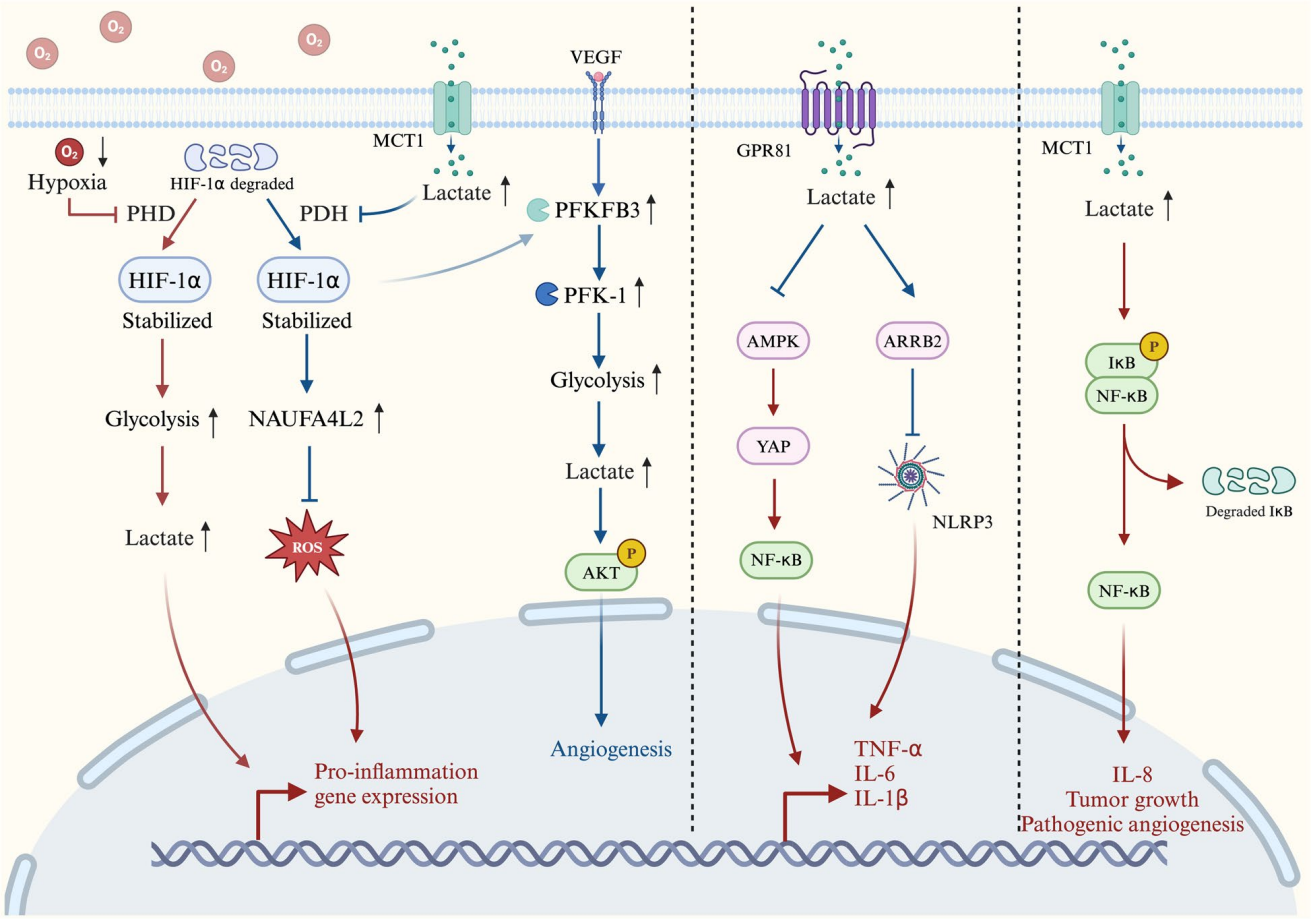
Lactate signalling pathways in the inflammatory response. *The Lactate/HIF-1α pathway*. In the early stage of inflammation, PHD activity is inhibited under hypoxic conditions and HIF-1α stabilizes, promotes glycolysis to produce lactate, and promotes proinflammatory gene expression. With progression of the inflammatory response, accumulated lactate inhibits pyruvate dehydrogenase. Stabilized HIF-1α can activate both the HIF-1α/PFKFB3 and HIF-1α/NDUFA4L2 signalling pathways, promoting angiogenesis and decreasing pro-inflammatory gene expression by limiting ROS production. *The Lactate/GPR81 pathway.* Activation of the lactate/GPR81 pathway inhibits either the AMPK/NF-κB or ARRB2/NLRP3 signalling pathways and reduces the production of TNF-α, IL-6, IL-1β, and NLRP3. *The Lactate/NF-κB pathway.* Intracellular lactate accumulation through MCT1 transport triggers the phosphorylation of IκBα, stimulates NF-κB, and induces IL-8 expression, thereby promoting cell migration and angiogenesis.

Lactate, a pivotal signalling molecule, plays a crucial role in orchestrating signal transmission among diverse cells, organs, and tissues. The lactate shuttle in inflammatory tissue environments is driven by concentration or pH gradients or reduction‒oxidation states, thereby facilitating the subsequent activation of pertinent signalling pathways that regulate tissue energy metabolism and immune function. The lactate signalling pathways involved in the inflammatory response are shown in Fig. 2.

#### HIF-1α

HIF-1 is an oxygen-dependent transcriptional activator composed of HIF-1β subunits and either HIF-1α, HIF-2α, or HIF-3α subunits [84]. The transcription factor HIF-1α is the pivotal mediator of the hypoxic response. Under hypoxic conditions, the activity of prolyl hydroxylase (PHD) is suppressed, leading to the stabilization and nuclear translocation of HIF-1α for its binding with hypoxic regulatory genes. The HIF-regulated genes, which are activated through Toll-like receptors (TLRs) and NF-κB, function in concert to promote the metabolic shift to anaerobic glycolysis and balance the cellular pH in response to increased lactic acid production. Consequently, increased HIF activity supports the innate immune functions of phagocytic cells by promoting phagocytosis, inhibiting apoptosis, and stimulating the release of antimicrobial peptides, granule proteases, and proinflammatory cytokines [85].

In hypoxic and hypermetabolic tissues, such as those seen in inflammatory conditions, upregulated expression of LDH-A and PDK1, which are direct transcriptional targets of HIF and are highly inducible by hypoxia, inhibit PDH and stabilize HIF-1. HIF-1 shifts metabolism from oxidative to glycolytic, thereby reducing pyruvate entry into the TCA cycle and oxidative metabolism [86, 87]. The HIF-1α/PFKFB3 pathway is activated, and HIF-1α highly expresses PFKFB3, which further upregulates F-2,6-BP expression via vascular endothelial growth factor (VEGF) signalling. Subsequently, F-2,6-BP is formed to activate PFK-1 [88], which alleviates the inhibition of PFK-1 by ATP and increases glycolytic flux [89]. Activated endothelial cells produce a substantial amount of lactate that induces protein kinase B phosphorylation to promote the proliferation of vascular endothelial cells [90]. Similarly, lactate, which is produced by activated dendritic cells, stabilizes HIF-1α and induces the expression of the NADH dehydrogenase (ubiquinone) 1 α subcomplex 4-like 2 (NDUFA4L2). NDUFA4L2 is a subunit of respiratory complex I that limits the activity of this complex and the generation of mitochondrial ROS, which is a byproduct of complex I. The activated HIF-1α/NDUFA4L2 pathway restricts mitochondrial respiration in DCs by repressing the increase in basal respiration and proton leakage. Thus, the expression of proinflammatory cytokines decreases in DCs by limiting the production of mitochondrial ROS, which activate the transcription factor XBP1 [91].

#### G protein-coupled receptor 81 (GPR81)

Lactate selectively activates GPR81 within the concentration range of 0.1–30 mM [92], thus functioning as an autocrine and paracrine signalling molecule [93] that is involved in regulating energy metabolism and inflammation. The expression level of GPR81 is relatively high in adipose tissue but low in other organ tissues [94]. Under inflammatory conditions, hypoxia promotes yes-associated protein binding to HIF-1α, leading to increased expression of glycolysis-stimulating proteins or enzymes [95]. The lactate/GPR81 pathway suppresses yes-associated protein activation and nuclear translocation through adenosine monophosphate-activated protein kinase (AMPK) phosphorylation, thereby attenuating NF-κB activation and subsequently reducing the LPS-induced production of the proinflammatory cytokines TNF-α and IL-6 in macrophages; this suppression ultimately leads to inhibition of the macrophage inflammatory response to LPS stimulation [96].

The anti-inflammatory effect of the lactate/GPR81 pathway also depends on arrestin β-2 (ARRB2), which is the adapter protein of GPR81. Activation of the lactate/GPR81 pathway induces the expression of the intracellular adaptor β-arrestin 2 signalling molecules, thus restraining Toll-like receptor activation of the nucleotide-binding oligomerization domain, leucine-rich repeat and pyrin domain containing 3 (NLRP3) inflammasome formation and IL-1β production, mitigating the inflammatory response, and minimizing inflammation-associated tissue damage in mice [97, 98].

#### NF-κB

NF-κB is a complex protein system consisting of transcription factors that govern the expression of genes involved in innate and adaptive immunity, inflammation, oxidative stress response, and B-cell development [99]. Composed of p50 and RelA/p65 subunits, NF-κB exists as a heterodimer in an inactive form in complex with the inhibitor of nuclear factor kappa B (IκB) in the cytoplasm [100]. In the inflammatory microenvironment, inflammatory cytokines, pathogen-associated molecular patterns, or antigen/immune stimulatory signals activate the IκB kinase complex, leading to the phosphorylation, ubiquitination, and degradation of IκB, thereby activating NF-κB.

Lactate, transformed from tumour cells through the MCT4 into ECs through the MCT1, induces an increase in ROS production, and then triggers the phosphorylation and consecutive degradation of IκBα. The lactate/NF-κB pathway, which is stimulated by IκBα degradation, subsequently induces IL-8 expression, thus supporting angiogenesis and tumour growth [90].

As mentioned above, the regulatory effect of lactate on the inflammatory response is dynamic. Specifically, under physiological conditions, in the acute inflammatory response, HIF-1α promotes the metabolic reprogramming of immune cells to promote glycolysis and the production of lactate, thereby promoting the inflammatory response by inducing NF-κB expression. In the chronic inflammatory response, lactate accumulates in large amounts, and the lactate/GPR81 pathway inhibits either the AMPK/NF-κB or ARRB2/NLRP3 signalling pathway to promote resolution of the inflammatory response. However, under pathological conditions, such as in tumours, lactate can lead to prolonged inflammation and pathological angiogenesis.

### Functions of lactate in diverse types of cells

**Fig. 3:**
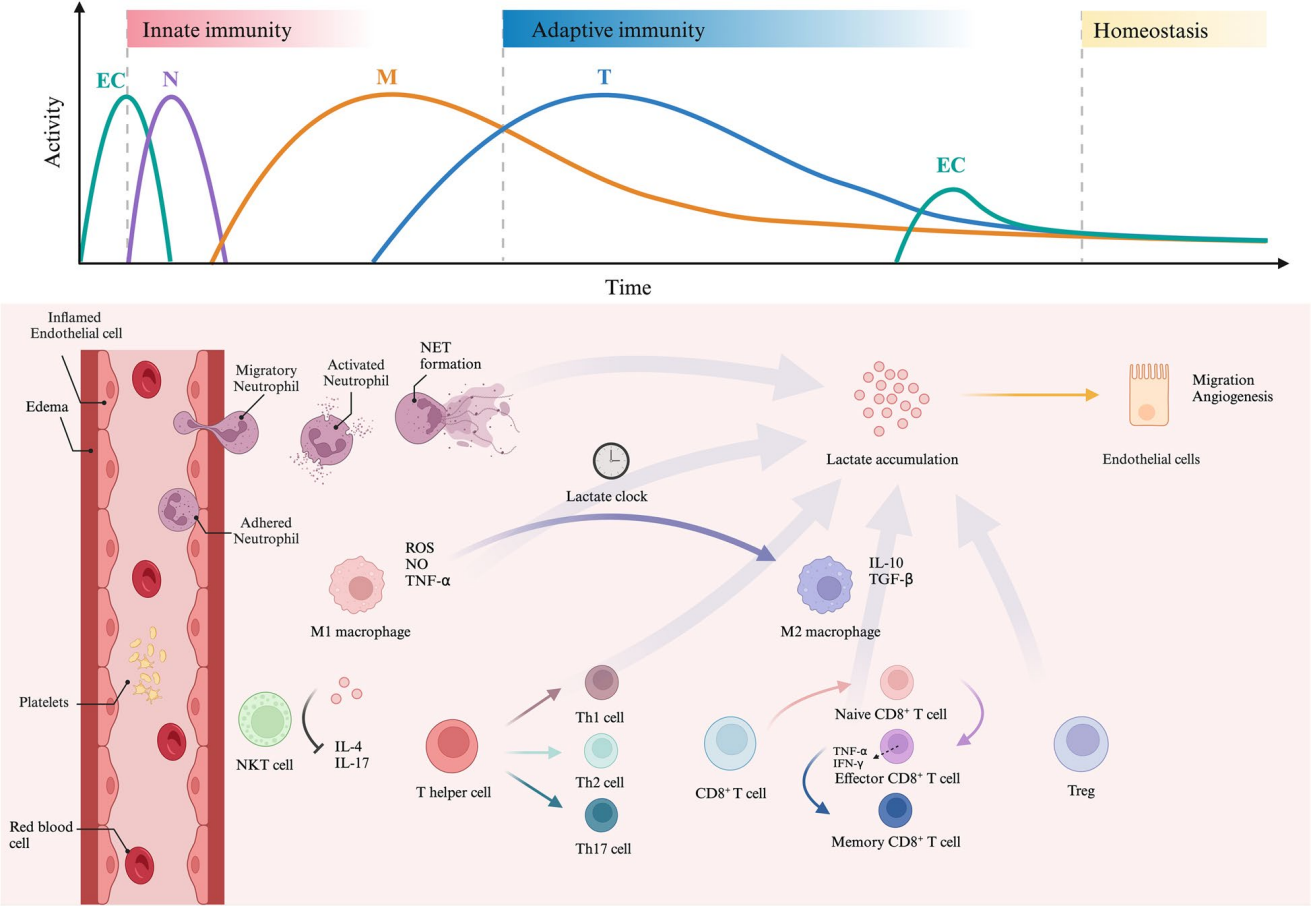
Lactate participates in inflammatory responses and regulates the functions of immune cells. The inflammatory processes involving inflammatory immune cells are highly dynamic. ECs are initially activated and express cell adhesion molecules, which facilitate the adherence of immune cells to the vascular wall, increase vascular permeability, and contribute to edema. Neutrophils are rapidly recruited to the site of infection for ROS generation and the formation of neutrophil extracellular traps. Macrophages are then polarized into M1-like macrophages, leading to a proinflammatory response. As lactate accumulates, the ‘lactate clock’ turns. Lactylation induces M1-like macrophages to polarize into M2-like macrophages through epigenetic mechanisms. T cells successively differentiate into CD4^+^ T cells and CD8^+^ T cells. Accumulated lactate inhibits the production of IL-4 and IL-17 in NKT cells and promotes the differentiation of CD4^+^ T cells into Th1, Th2 and Th17 cells. Subsequently, the transformation of cell energy metabolism to glycolysis promotes the differentiation of naive CD8^+^ T cells into effector CD8^+^ T cells, which secrete TNF-α and IFN-γ. Eventually, T cells transform into Treg cells and memory CD8^+^ T cells in the resolution of inflammation. Moreover, the accumulation of lactate, which is produced by various immune cells, induces EC migration and angiogenesis, thereby helping maintain inflammatory homeostasis.

In response to injury, resident immune cells become activated, initiate EC proliferation, and recruit additional immune cells to the site of injury. Following the resolution of inflammation, M2 macrophages eliminate activated immune cells and facilitate the generation of regulatory and memory T cells, thereby inducing immune tolerance and suppressing inflammation [101]. The proper functioning and collaboration among these cell types necessitate glycolysis activation to ensure a timely supply of ATP and lactate, as depicted in Fig. 3.

#### Vascular cells

The inflammatory response initially triggers the activation of ECs, leading to the production of NO, which in turn induces the expression of cell adhesion molecules, including E-selectin, intercellular adhesion molecule 1, and vascular cell adhesion molecule 1. These adhesion molecules facilitate the adherence of immune cells to the vascular wall, increase vascular permeability, and consequently contribute to interstitial oedema [102]. Under physiological conditions, ECs predominantly rely on glycolysis for energy production and exhibit significantly greater glycolytic activity than other healthy cells [103]. This unique cellular metabolism results in the extensive production of lactate, which is taken up by pericytes and plays a crucial role in pericyte metabolism, including energy generation and amino acid biosynthesis [104]. The overexpression of HIF-1α in inflammatory and hypoxic environments further promotes glycolysis in ECs and induces the expression of cell adhesion molecules, proinflammatory cytokines, and chemokines [105]. Moreover, HIF-1α induces the expression of PFKFB3 via VEGF, leading to an increase in glycolysis and the production of a large amount of lactate in ECs [89].

ECs also participate in post-inflammatory tissue repair under the induction of lactate. This process facilitates the proliferation and migration of ECs through protein kinase B phosphorylation and regulates the balance between coagulation and fibrinolysis [89]. In patients with severe sepsis, the persistent destruction of ECs induced by inflammation leads to the recruitment and migration of activated leukocytes, thereby inducing a prothrombotic and antifibrinolytic state in the body [106]. This state may contribute to the development of inflammatory diseases, including microvascular thrombosis, organ ischaemia, and multiple organ dysfunction syndrome [107].

#### Innate immune cells

Neutrophils, accounting for approximately 60% of leukocytes in the blood circulation, play a crucial role in innate cellular immunity [108]. During infection, neutrophils are rapidly recruited to the site of infection, where they execute diverse functions, including microbial eradication, phagocytosis, the generation of ROS through oxidative bursts, and the formation of neutrophil extracellular traps [109]. The inflammatory response promotes an increase in glycolytic activity within neutrophils, resulting in an increase in the production and release of lactate [110]. This metabolic shift further promotes phagocytosis and the formation of neutrophil extracellular traps [111, 112]. Lactate triggers GPR81 receptor activation, resulting in increased vascular permeability, as well as elevated levels of chemokine (C-X-C motif) ligand 1 and (C-X-C motif) ligand 2. Additionally, lactate promotes the release of cytokine granulocyte colony-stimulating factor to induce neutrophil generation [113].

Macrophage polarization is a highly dynamic process that occurs during inflammatory diseases and requires an increase in glycolytic flux to rapidly generate adequate amounts of ATP and supply lactate as a carbon substrate [114]. Activated macrophages can be categorized into two phenotypes, M1-like and M2-like, based on their polarization state. M1-like macrophages primarily rely on glycolysis for the generation of ROS and NO and release proinflammatory cytokines such as TNF-α, IL-1β, IL-12, and IL-23, leading to a proinflammatory response and pathogen elimination. Conversely, M2-like macrophages rely on fatty acid oxidation and the TCA cycle to induce the production of polyamines and L-proline, along with the secretion of IL-10 and transforming growth factor β, which are involved in immune regulation and tissue repair processes [115]. The induction of macrophage polarization by lactate shows obvious time series characteristics. Lactate can not only stimulate the expression of proinflammatory genes in the early stage of inflammation but also have a synergistic effect with other proinflammatory substances on the expression of proinflammatory genes [116]. However, as lactate accumulates, the process of lactylation initiates the regulation of gene transcription and triggers M2-like macrophage polarization in a time-dependent manner, referred to as the ‘lactate clock’, thereby regulating the inflammatory response. In the late phase of inflammation, lactylation induces the transformation of M1-like macrophages into M2-like macrophages through epigenetic mechanisms, thus helping repair tissue damage caused by inflammation [19].

#### Adaptive immune cells

Natural killer T (NKT) cells are lymphocytes that possess characteristics of both innate and adaptive immune cells and serve as a link between the two immune responses. Compared with conventional T cells, NKT cells demonstrate accelerated initiation of effector functions upon activation but exhibit diminished metabolic activity in peripheral organs. Although NKT cells promote mTORC signalling and glycolysis upon encountering antigens, the presence of a high lactate microenvironment impairs NKT cell homeostasis and effector function, thus concurrently inhibiting the production of the proinflammatory cytokines IL-4 and IL-17. Consequently, NKT cells primarily rely on glutamine as the primary source of carbon for survival and proliferation through OXPHOS [117].

Lactate functions as a fuel for the TCA cycle and cellular biosynthesis, particularly during functional T-cell responses [15]. Inactivated T cells primarily rely on OXPHOS for ATP production and exhibit a less metabolically active state than activated T cells. Similarly, the activation of CD4^+^ T cells is solely dependent on OXPHOS. However, activated CD4^+^ T cells increase aerobic glycolysis and lactate production to fuel proliferation by upregulating HK2, LDHA, and MCT4 expression and downregulating pyruvate dehydrogenase kinase 1 expression [47, 118]. During the inflammatory response, CD4^+^ T cells differentiate into effector CD4^+ ^T cell subsets, such as Th1, Th2 and Th17 cells, or regulatory T cells (Tregs), with effector T cells contributing to the immune response by producing proinflammatory cytokines, whereas Treg cells suppress immunity and inflammation. Furthermore, the functional metabolism of effector T cells undergoes a shift towards reliance on OXPHOS and aerobic glycolysis [119]. In chronic inflammatory tissues, extracellular lactate downregulates HK1 expression via MCT1, reduces glycolytic flux, and inhibits the migration of T cells to retain them at the site of inflammation. Simultaneously, elevated levels of exogenous lactate can activate the expression of the transporter SLC5A12 to facilitate lactate entry into CD4^+^ T cells, promote IL-17 production, and reduce cell lysis via the PKM2/STAT3 signalling pathway to sustain chronic inflammation [33]. The metabolic profile of Treg cells differs from that of effector T cells. Treg cells take up more lactate, which is converted into pyruvate, to produce citrate and malate, thereby fuelling the TCA cycle. Malate is converted to oxaloacetate and subsequently to phosphoenolpyruvate to fuel gluconeogenesis, promote Treg cell proliferation, and maintain immunosuppression [18].

TCR signal transduction activates CD8^+^ T cells to induce glucose uptake and the transformation of cell energy metabolism to glycolysis; TCR signal transduction also promotes the activation, proliferation, and differentiation of naive CD8^+^ T cells into effector CD8^+^ T cells and induces the secretion of TNF-α, IFN-γ, and other proinflammatory cytokines to exert immune effects [120]. Increased glycolysis is not conducive to the long-term survival of memory cells, so the energy metabolism of memory T cells is reconverted to OXPHOS, which is fuelled by fatty acid oxidation [121]. However, compared with naive CD8^+^ T cells, memory CD8^+^ T cells exhibit faster induction of glycolysis, which facilitates rapid cellular proliferation, effective adaptive immune responses, and timely cell apoptosis [120].

## Roles of lactate in inflammation

### Role of lactate in acute inflammation

During acute inflammation, the metabolic transition from OXPHOS to aerobic glycolysis expedites energy production by converting pyruvate to lactate. Moreover, this conversion provides metabolic intermediates, i.e., lactate, to support cell proliferation, cytokine production, and anti-inflammatory responses [122]. The acute inflammatory response is a self-limiting mechanism that is employed by the host for protection, and its initiation and resolution are tightly regulated by proinflammatory and anti-inflammatory mediators. The excessive release of inflammatory mediators into the bloodstream can result in sepsis and tissue damage, eventually leading to fatal consequences [123]. Macrophages can absorb lactate, which is found at abnormally elevated levels in the blood of septic patients, resulting in an increase in intracellular lysine lactylation (Kla) of the high mobility frame 1 (HMGB1) protein. Subsequently, lactylated HMGB1 is excreted and released via the exosome pathway, thereby disrupting endothelial integrity, increasing vascular permeability, and impairing endothelial barrier function [37]. Ultimately, this process facilitates the progression of sepsis. The lactate level is a sensitive, independent indicator of cellular and metabolic stress [124]. Elevated lactate levels may serve as a crucial indicator of 'cryptic shock'. The combination of hyperlactatemia and fluid-resistant hypotension provides a more reliable approach to identify the physiological and epidemiological signs of septic shock; the utilization of this combination surpasses that of any single criterion alone [125]. Lactate clearance is significantly reduced in patients with septic shock, and persistent hyperlactatemia or even elevated lactate levels may reflect reduced clearance rather than increased lactate production [126].

IL-33 can increase IgE-induced inflammation and the level of IL-33 is elevated in patients with acute allergic diseases such as asthma and atopic dermatitis. Lactate can induce the production of IL-33, and a reduced rate of glycolysis can inhibit IL-33 signalling, thereby preventing neutrophil recruitment and mast cell activation in vivo and limiting the acute inflammatory response [127].

### Role of lactate in chronic inflammation

Unlike acute inflammation, chronic inflammation can persist and inflict tissue damage as a result of enduring irritants and the inability to resolve the inflammatory response. Lactate is one of the most abundant byproducts of cellular metabolism in inflammatory tissues. The impact of short-term exposure to lactate on the release of cytokines from tissues is limited. However, long-term exposure can mediate immunoinflammatory responses [128]. The accumulation of lactate amplifies the inflammatory response in chronic inflammatory diseases and leads to the retention of CD4^+^ and CD8^+^ T cells, resulting in elevated levels of the proinflammatory cytokine IL-17 and impaired cytolytic activity [129]. This process contributes to the development of chronic inflammation, ultimately inducing tissue damage [33]. The activation of proinflammatory microglia is a characteristic feature of Alzheimer's disease. The increase in Kla levels in β-amyloid plaques activate the transcription of multiple genes encoding glycolytic enzymes, thereby exacerbating neuroinflammation [130]. Injection of lactate into the extracellular space acts as a negative feedback signal that restricts excessive inflammatory responses mediated by glycolysis-promoting immune cells [128].

Previous studies have indicated that the inflammatory response plays a crucial role in the initiation and progression of tumours by facilitating tumour cell proliferation, survival, and migration [131, 132]. In mouse xenograft models of human colorectal and breast cancer, lactate released by tumour cells stimulates IL-8 production in ECs through the MCT4 transporter protein, thereby promoting tumour growth and angiogenesis [90]. Lactate also significantly influences immune regulation by upregulating the expression of PD-L1 through GPR81, suppressing T-cell effector functions, reducing IFN-γ production, and promoting tumour proliferation and metastasis [133]. Metformin is a double-edged sword in the treatment of tumours [134]. Research shows that when using metformin alone, high doses may be required to see inhibitory effects on tumours [135], but high-dose administration of metformin may inhibit OXPHOS by targeting mitochondrial complex I (NADH dehydrogenase), leading to the accumulation and conversion of NADH and pyruvate into lactic acid and resulting in lactic acidosis in cancer cells, which promotes cancer progression. Blocking the lactate/GPR81 pathway with metformin can effectively inhibit glycolysis and decrease lactate levels within tumours, thereby increasing the number of CD8^+^ T cells in tumour tissue and IFN-γ secretion in lymph nodes. Subsequently, dual blockade of the lactate/GPR81 pathway and the PD-1/PD-L1 pathway significantly inhibited tumour growth and induced tumour regression [136]. Furthermore, within the tumour microenvironment, lactate can induce the secretion of hyaluronic acid and VEGF, thereby facilitating tumour metastasis and proliferation [137, 138]. In chronically inflamed tissues, this effect could be utilized to promote EC migration and tissue repair.

### Regulation of the inflammatory response by lactylation

When lactate accumulates to a certain threshold within cells, the 'writer' p300 enzyme transfers lactyl-CoA to histone lysine residues, thus modulating the interaction between histones and DNA to generate this posttranslational modification. This regulatory process is known as lactylation [139]. Specifically, under hypoxic conditions such as inflammation, M1 macrophages undergo metabolic reprogramming that is characterized by increased glycolysis and suppressed TCA cycle activity, resulting in an increase in lactate production. Subsequently, lactyl-CoA synthetase converts lactate to lactyl-CoA, thereby inducing arginase 1 and Vegfa expression via histone Kla. Approximately 16–24 h later, when cellular lactate levels reach a specific threshold, the ‘lactate clock’ is activated to induce Kla expression on histones, thus gradually inducing polarization of M2-like macrophages. As a result, M2-like macrophages secrete considerable amounts of IL-10 and transforming growth factor β to suppress inflammatory responses, which contribute to tissue repair, favour tissue remodelling, initiate angiogenesis, and preserve tissue homeostasis [19, 115].

Epigenetic alterations in the intense during immunoinflammatory responses such as sepsis will impair the expression of genes that regulate crucial immune activation responses, thereby rendering the host more susceptible to infection [140]. Overactivation or inadequate regulation of macrophages during the early stages of sepsis can lead to an inflammatory storm [141]. Most sepsis patients will progress to an immunosuppressive state after the early hyperinflammatory phase, and due to lymphocyte exhaustion and reprogramming of the function of innate immune cells, the longer sepsis continues, the greater the likelihood of developing immunosuppression; immunosuppression can lead to a greater incidence of infections and multiple-organ dysfunction, which often account for late mortality [142–144]. An expert consensus on the monitoring and treatment of sepsis-induced immunosuppression recommends that the rapid detection of epigenetic changes, the early identification of immunosuppressive states and timely treatment to enhance immune capacity can help prevent persistent immunosuppression and reduce mortality [145]. The regulation of macrophage polarization during the destructive phase of the inflammatory response has been recognized as a key factor in sepsis pathology [146]. Refraining the release of inflammatory factors by monitoring and regulating the level of lactylation may be a potential strategy to reduce tissue damage and mortality caused by destructive inflammatory reactions.

## Lactate-related therapy for inflammation-related diseases

Lactate is elevated in many disease conditions and appears to have contradictory effects. In the acute stage of inflammation, appropriately increasing glycolytic flux is necessary to rapidly acquire energy. However, excessive lactate production may result in extracellular acidosis and uncontrolled inflammation, thereby exacerbating injury. Conversely, in the late stage of inflammation, a substantial increase in lactate can effectively suppress an exaggerated inflammatory response and expedite tissue recovery. Lactate and its metabolites can serve as carbon sources to fuel the TCA cycle or OXPHOS during the late stage of inflammation, thereby enhancing metabolic adaptations during recovery and suppressing inflammatory responses by memory T cells and Tregs [18, 147].

**Fig. 4:**
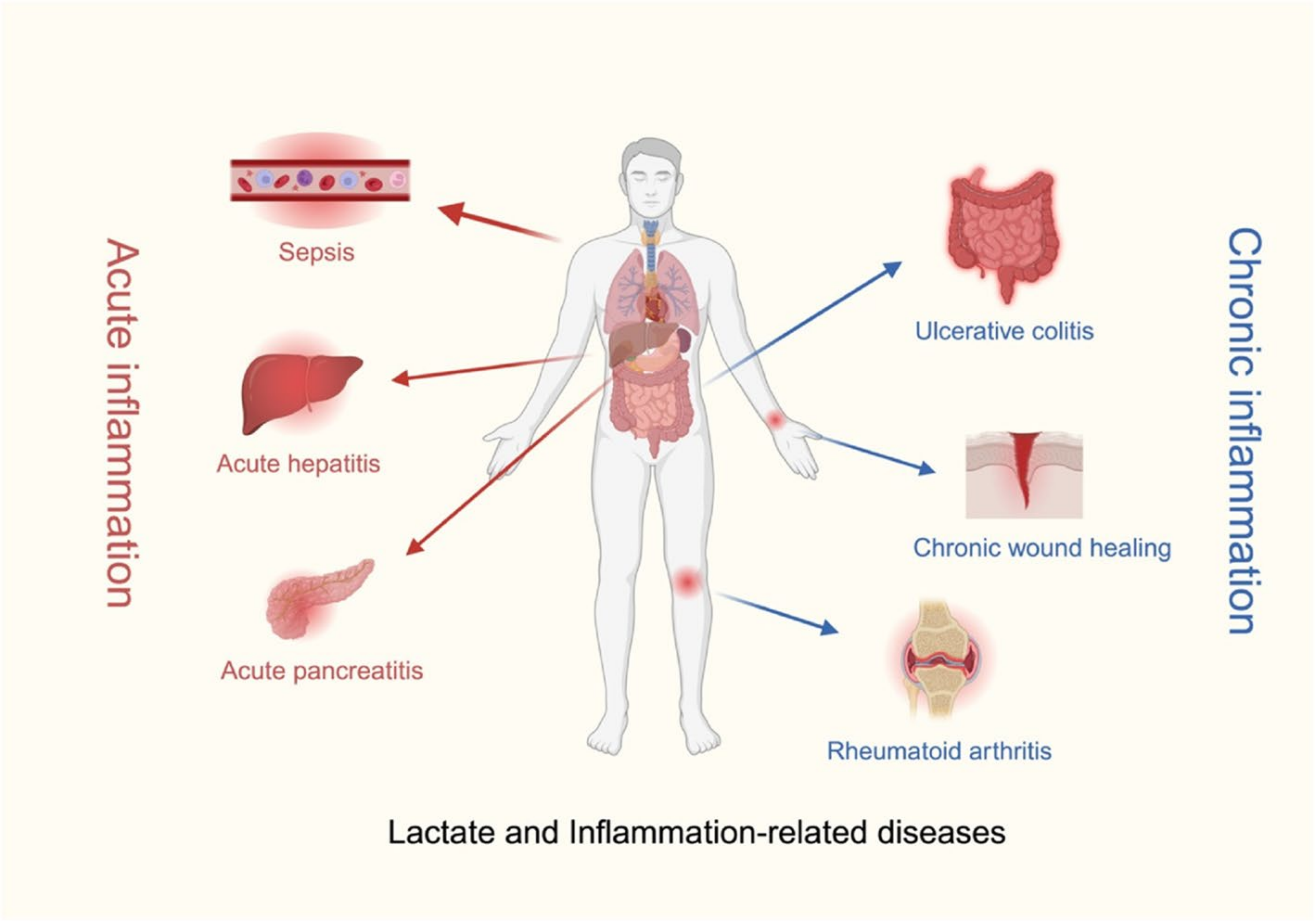
Lactate and inflammation-related diseases. Lactate-related therapy is widely used for acute inflammatory diseases (sepsis, acute hepatitis, and acute pancreatitis) and chronic inflammatory diseases (ulcerative colitis, rheumatoid arthritis, and chronic wound healing). The red arrows represent acute inflammation, and the blue arrows represent chronic inflammation.

Due to the important role of lactate in the occurrence and development of various inflammatory diseases, the application of lactate-related therapies has been proposed to improve disease outcomes in patients with inflammatory diseases; these therapies have effects such as increasing buffering capacity and regulating lactate transporter expression. The impact of lactate on inflammation provides novel therapeutic approaches for numerous inflammation-related diseases, making lactate-related therapy indispensable and efficacious for previously challenging to treat conditions [148]. Lactate and inflammation-related diseases are shown in Fig. 4.

### Lactate-related therapy for acute inflammatory diseases

Serum lactate levels have significant clinical implications in monitoring the progression of acute inflammatory diseases, such as acute peritonitis, acute pancreatitis, sepsis, and septic shock [149]. Thus, lactate metabolism may be a potential target for immunomodulatory intervention in patients suffering from acute inflammatory diseases.The Third International Consensus Definitions for Sepsis and Septic Shock recommended that serum lactate levels > 2 mmol/L (18 mg/dL) be added to the diagnostic criteria for septic shock [125]. Hyperlactemia is often a physiological response to mitigate the impact of injury. While it may confer protection during the initial cytokine storm, it can become pathogenic in the late stage of sepsis, due to the anti-inflammatory effects mediated by lactylation. H3K18 lactylation is greater in patients with septic shock than in patients without septic shock, and the levels of procalcitonin and inflammatory factors are positively correlated [150]. This correlation indicates that lactylation caused by lactate accumulation may be involved in the pathophysiology of septic shock. Either inhibiting endogenous lactate production by decreasing glycolytic flux or blocking extracellular lactate uptake could attenuate HMGB1 lactylation in macrophages, thereby alleviating the side effects of sepsis [37]. These findings suggest that lactate not only serves as a viable therapeutic target for the immunopathology of sepsis but also functions as a readily measurable and repeatable diagnostic biomarker for sepsis; thus, lactate could play a role in the treatment of sepsis [151].

Inhibition of the lactate transporter SLC5A12 decreased peritoneal lactate levels to reestablish T-cell migration away from inflammatory sites and blocked the production of proinflammatory cytokines in mice with glycan-induced peritonitis [129]. Lactate negative regulates the TLR-induced NLRP3 inflammasome and IL-1β production through GPR81. Intraperitoneal injection of lactate reduces inflammation and organ damage in mice with immune hepatitis. Additionally, subcutaneous injection of lactate after tissue injury successfully reduced the severity of acute pancreatitis and acute liver injury in mouse models [152]. Relevant clinical studies have also shown that lactate can reduce the serum amylase concentration, ameliorate pancreatic lesions, and reduce the severity of acute pancreatitis and acute liver injury.

### Lactate-related therapy for chronic inflammatory diseases

Although the development of modern medicine has greatly reduced the rate of mortality associated with acute inflammation, the prevalence of chronic inflammatory diseases has also increased [153]. Chronic inflammation is defined as the long-term result of chronic physiological stimulation of the innate immune system that occurs later in life and has been recognized as one of the ‘seven pillars of ageing’ [154].

Researchers have shown that in patients with ulcerative colitis, the level of lactate secreted by intestinal mucosal cells is proportional to the severity of inflammation [155, 156]. Knockout of the lactate receptor GPR81 in mice with colitis increased inflammatory cytokine secretion in intestinal dendritic cells and macrophages, which worsened colon inflammation. In contrast, activation of GPR81 promotes immune regulation, induces the differentiation of CD4^+^ T cells to produce IL-10, delays disease onset, and reduces colon inflammation [157]. Intrarectal therapy with lactate can hinder the proinflammatory response of ECs, prevent histopathological damage, reduce bacterial translocation, and prevent intestinal inflammation [158]. The microenvironment of affected joints in patients with rheumatoid arthritis is characterized by hypoxia, resulting in the upregulated expression of glycolytic enzymes such as HK2 and LDHA [159]. This metabolic shift towards glycolysis disrupts the delicate balance between glycolysis and OXPHOS, leading to metabolic dysregulation and lactate accumulation, thereby catalysing the progression of rheumatoid arthritis [160, 161]. Intra-articular injection of adenovirus carrying murine HK2 into the knee joint of normal model mice can significantly increase synovial intimal thickness, increase the proliferation of activated fibroblast-like synovial cells, and reduce its invasive phenotype, thereby reducing the disease severity of arthritis [162].

Physiological wound healing is divided into four consecutive stages: haemostasis, inflammation, proliferation, and remodelling [163]. Chronic inflammation prolongs the inflammatory stage, cell proliferation, and tissue remodelling, which prevents wound healing and leads to the formation of chronic wounds. If the wound takes more than 2 weeks to remodel, hyperplastic scars will develop [164]. Recent studies have demonstrated that chronic wounds exhibit a persistent low-grade proinflammatory state that is characterized by a significantly greater abundance of M1 macrophages than M2 macrophages [165, 166]. This imbalance leads to the establishment of an excessive inflammatory environment, in which excessive inflammatory mediators, i.e., TNF-α and IL-1β, continue to destroy wound tissue [167]. However, the levels of cytokines and growth factors contributing to healing, i.e., TGF-β and VEGF, are reduced in chronic wounds [168]. As a highly efficient energy source, lactate can meet the high metabolic demand of various growth and cytokine production processes and provide a supply of energy to cells during wound healing. During wound healing, reduced carbon dioxide levels and high oxygen tension can increase pH, which impedes the division and proliferation of fibroblasts and keratinocytes, as well as skin transplantation [169]. Conversely, the increased capacity for oxygen delivery and reduced protease activity in neutral or even acidic environments can promote wound healing [170]. By increasing the concentration of lactate in local tissue, HIF-1α can be stabilized even under aerobic conditions, and subsequently, M2 polarization of macrophages is induced [166]; this polarization facilitates VEGF generation to drive neovascularization and expedite wound healing [171–173].

## Lactate-based strategies for the management of inflammation-associated disorders

Inflammatory tissues can produce the ‘Warburg effect’, stimulating aerobic glycolysis to promote ATP generation and the accumulation of lactate as a crucial carbon source [25], thereby meeting the anabolic demands of the activation and function of immune cells. Thus, the regulation of glycolytic enzymes is one of the possible therapeutic strategies for the treatment of inflammation-related diseases. 2-deoxy-D-glucose can inhibit HK2, the first rate-limiting enzyme in glycolysis, to reduce the production of T-cell-dependent autoantibodies, the proliferation of B cells, and the migration of dendritic cells [174]. The use of 2-deoxy-D-glucose in the treatment of arthritic model mice and systemic lupus erythematosus model mice hindered the inflammatory response and reduced the number of Th17 cells [47, 48]. The PKM2 dimer can interact with Hif-1α and increase the expression of proglycolytic enzymes [175]. Tetramerization of the PKM2 monomer/dimer counteracts the LPS-induced increase in glycolysis and modulates the host immune response in vivo by attenuating IL-1β production and promoting IL-10 secretion, leading to an increase in bacterial transmission and impaired infection clearance [176]. Targeted inhibition of PKM2 and a reduction in lactate production can prevent the initiation of eukaryotic factor 2 α kinase 2 phosphorylation and inflammasome activation, thereby inhibiting the release of IL-1β, IL-18, and HMGB1 from macrophages and reducing the mortality of septic mice [62, 63]. TEPP-46, a small molecule PKM2 activator that induces PKM2 tetramerization and blocks PKM2 nuclear translocation, can inhibit the proliferation of Th1 and Th17 cells, reduce the severity of encephalomyelitis, and reverse lactate-induced IL-17 production [177]. However, Seki et al. reported that TEPP-46 not only restricts the differentiation of Th17 cells but also impedes the generation of Tregs, which are anti-inflammatory T cells, by disrupting signalling pathways triggered by TGF-β [178]. These findings suggest a need to re-evaluate the therapeutic potential of PKM2 activators. AdPFKFB3 transfection can improve the viability of human chondrocytes, reduce the activation of caspase 3, and promote the expression of aggregator and type II collagen [179]. Targeted knockout of PFKFB3 leads to a significant reduction in EC glycolysis and effectively inhibits the NF-κB and HIF-1α signalling-mediated endothelial inflammatory response, thereby impeding the onset and progression of pulmonary hypertension, sepsis, and acute lung injury in murine models [180, 181]. Targeted inhibition of pyruvate dehydrogenase kinase 1 by dichloroacetate inhibited disease progression in mouse models of colitis and experimental autoimmune encephalomyelitis by inhibiting glycolysis, promoting oxidative metabolism and ROS production, and limiting the proliferation and function of Th17 cells [182]. LDHA is the key enzyme that induces the conversion of pyruvate into lactate during glycolysis, and inhibitors such as sodium oxalate and diclofenac can effectively block the production of lactate in mouse models; these inhibitors increase anti-inflammatory capacity of immune cells and prevent inflammation [83, 183]. Targeted inhibition of MCT4 (phloretin, α-cyano-4-hydroxycinnamate, and AR-C155858) and SLC5A12 (anti-Slc5a12 antibody and shRNAs) inhibits the motility of CD8^+^ and CD4^+^ T cells, respectively, by reducing lactate and lactic acid concentrations in the extracellular space, thus reducing disease severity in mouse models of rheumatoid arthritis [129].

Moreover, lactate acts as a signalling molecule in the inflammatory response and is constantly exchanged between producers (glycolytic cells) and consumers (oxidizing cells) to regulate immune cell function. In the inflammatory environment, lactate-related transporters such as MCTs, SMCTs, and GPR81 control lactate transport and regulate immune cell function through the HIF-1α, NF-κB, and other signalling pathways. Sanmarco LM et al. [91] designed a probiotic that can produce D-lactate to activate the HIF-1α/NDUFA4L2 signalling pathway and restrict ROS activation in DCs to regulate T-cell immune function. The activation of GPR81 by lactate at physiological concentrations can reduce oxidative stress and inhibit the production of proinflammatory cytokines such as IL-6, IL-8, monocyte chemoattractant protein, and HMGB1, which indicates that GPR81 could be a therapeutic target for endothelial inflammation [184]. Ketogenic diet therapy addresses the energy requirements of glaucoma patients and inhibits the inflammatory response by activating the lactate-GPR81 signalling pathway and inhibiting AMPK/NF-κB signalling and NLRP3 inflammasome activation [98]. Oral administration of a GPR81-specific agonist in a mouse model of colitis limited the differentiation of Th1 and Th17 cells and induced the differentiation of regulatory T cells to inhibit intestinal inflammation [157]. Furthermore, researchers have used 3,5-dihydroxybenzoic acid to target GPR81 expression in pregnant model mice, thus reducing endotoxin-induced uterine inflammation, the likelihood of premature birth, and related neonatal mortality [185]. Metformin, a nonspecific HK2 inhibitor, increased glucose uptake and reversed the effects of LPS-induced neuroinflammation in acute hippocampal slices by decreasing intra- and extracellular IL-1β and S100B levels [46]. Metformin is also an inhibitor of mitochondrial complex I, which can increase the rate of glycolysis and promote the production of lactate, thereby reducing TCA cycle activity and inducing the activation of AMPK. Subsequently, AMPK promotes the transformation of effector T cells into memory T cells and inhibits the development of immunoinflammatory diseases [186].

In addition, lactate accumulation activates the ‘lactate clock’ to induce lactylation and promotes cell proliferation and blood vessel formation. Several studies have demonstrated a strong association between lactylation and tumorigenesis, as well as cancer progression [187–189]. These results indicate that lactylation is a promising therapeutic target for innovative cancer interventions [190]. Cui H et al. [191] reported that histone lactylation can promote the expression of profibrotic genes in macrophages in patients with pulmonary fibrosis. After the targeted knockdown of p300, histone lactylation in macrophages decreases, and the proportion of macrophages with a profibrotic phenotype is reduced. Although promoting cell proliferation promotes the occurrence and development of diseases such as tumours and fibrosis, it is advantageous for patients whose wounds need to be healed, especially for target groups such as elderly people with delayed wounds and patients with chronic diseases. Lactylation may be a new epigenetic code that regulates inflammatory gene expression through the ‘lactate clock’ and is a potential target for promoting the healing of damaged tissues.

**Fig. 5:**
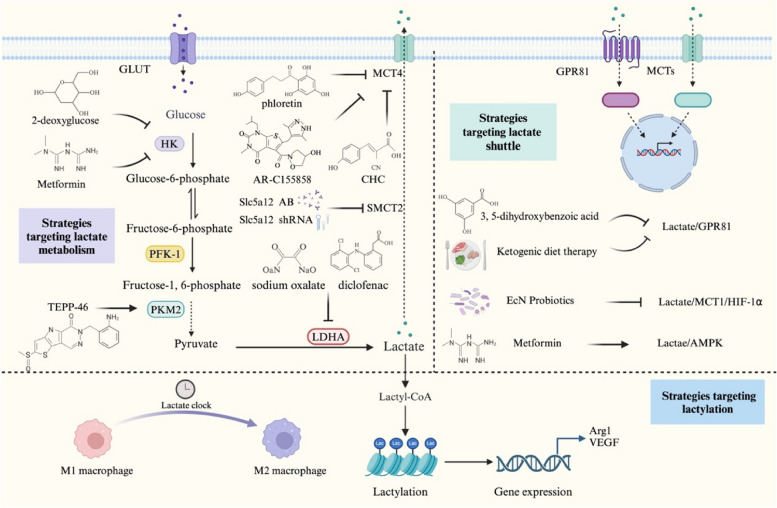
Strategies targeting lactate metabolism, the lactate shuttle, and lactylation to manage inflammation-related diseases. The unique role of lactate in metabolic feedback, signal transduction, and the posttranslational modification of proteins in inflammatory tissues makes it a valuable target for the treatment of inflammatory diseases.

Lactate is elevated in inflammatory diseases due to increased production or impaired clearance, which then influences immune cell function. The management of hyperimmune-mediated inflammatory conditions, such as acute hepatitis and pancreatitis [152], should prioritize the reduction of lactate production. Conversely, for immunosuppressive-related inflammatory diseases like tumours [192], the focus should shift towards promoting lactate clearance rather than inhibiting lactate production. Strategies targeting lactate metabolism, lactate shuttling, and lactylation to manage inflammation-related diseases are shown in Fig. 5.

## Conclusion

Inflammation is an adaptive response of the body to stress that is initiated by the immune system. The incidence of basic diseases gradually increases with age, and immune regulation gradually decreases [193], leading to an increase in the incidence and mortality of inflammation-related diseases [194]. Despite the obvious side effects of glucocorticoids, they are currently widely used in the clinic. As a result of efforts to develop more effective and less toxic drugs, biologics have emerged. The application of biologics has successfully improved the quality of life and extended the life span of countless patients, but biologics hinder host immune defence against infection and cancer [10]. Therefore, there is an urgent need to develop new drugs that are capable of mitigating organ toxicity and while minimizing their impact on infection and cancer immunity.

**Fig. 6:**
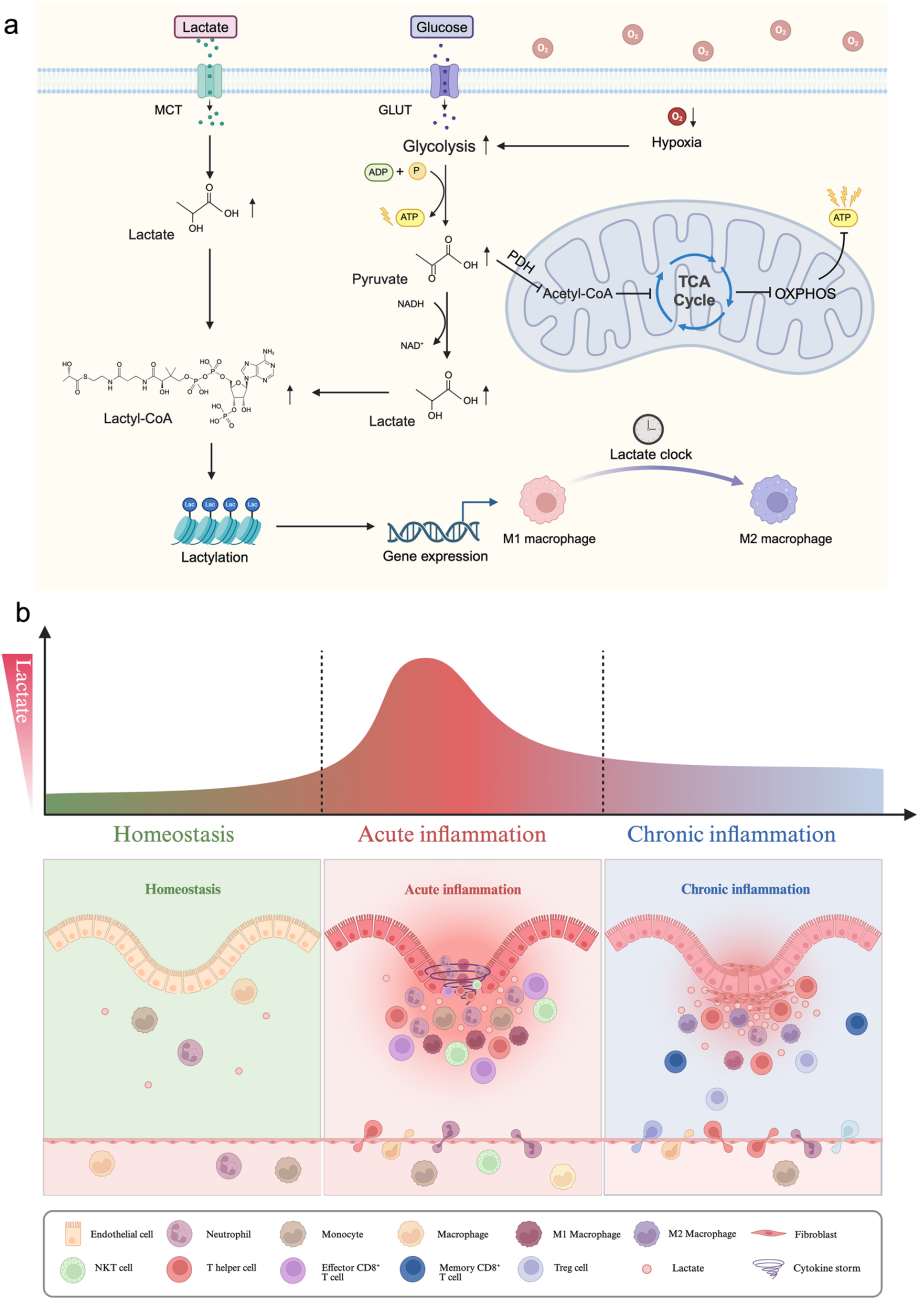
Schematic diagram showing the role of lactate metabolism in the inflammatory microenvironment at specific time points. **a** Lactate metabolism is implicated in the TCA cycle, lactylation, and epigenetic alterations based on the ‘lactate clock’ during the inflammatory response. Lactate can be generated intracellularly by glycolysis, or extracellular lactate can be transported in by MCT1. Following lactate accumulation, lactate is converted to lactoyl-CoA, which is involved in lactylation and drives epigenetic changes. **b** Lactate mediates the dynamic changes in inflammatory immune cells during different inflammatory periods. As a consequence of the lactate shuttle, lactate, which is transported by specific receptors, induces lactylation and epigenetic remodelling, alters various inflammatory immune cells, and exerts proinflammatory or anti-inflammatory effects in a dynamic time-dependent manner to help maintain inflammatory microenvironment homeostasis.

Currently, inflammatory diseases are treated by regulating lactate metabolism; for example, promoting the consumption of extracellular lactate, reducing the transport of lactate into immune cells, or directly blocking immune cell ingestion of lactate. Since lactate metabolism and the lactate shuttle play vital roles in normal tissues, directly blocking these processes may cause unwanted side effects. For instance, targeted inhibition of glycolytic enzymes to block lactate production directly leads to a decrease in the glycolytic rate and affects the energy supply and immune regulation. Lactylation is the most recently discovered contributor to the regulation of inflammation by lactate and is also a potential therapeutic target. The regulatory effect of lactylation on immunity, also called the ‘lactate clock’, links metabolism and gene regulation based on a time pattern [149], as presented in Fig. 6. Lactylation may provide new ideas and methods for the prevention and treatment of inflammation-related diseases.

In summary, the paradoxical role of lactate in the inflammatory diseases still needs to be explored. Moreover, research on the ‘lactate clock’ still needs to be performed to determine the proper level of lactylation required to regulate the inflammatory response at different periods. Counteracting the effects of acidity and preserving physiological lactate metabolism in immune cells to help resolve inflammation may be advantageous. Future studies on the use of lactate for the treatment of inflammatory diseases require long-term monitoring of the possible subsequent effects of this therapy on the body.

## Data Availability

No datasets were generated or analysed during the current study.

## Abbreviations

ATP: Adenosine triphosphate
NADH/NAD^+^: Nicotinamide adenine dinucleotide
TCA: Tricarboxylic acid
OXPHOS: Oxidative phosphorylation
LDH: Lactate dehydrogenase
HIF-1α: Hypoxia-inducible factor 1 α
MCT: Monocarboxylic acid transporter
PFKFB: 6-Phosphofructo-2-kinase/fructose-2,6-bisphosphatases
NF-κB: Nuclear factor kappa-B
HK2: Hexokinase 2
PFK-1: Phosphofructokinase-1
PKM: Pyruvate kinase M
IL: Interleukin
SMCT: Sodium-coupled monocarboxylate transporter
SLC: Solute carrier
Th: T helper cell
MAVS: Mitochondrial antiviral signaling proteins
PFK-2: 6-Phosphofructo-2-kinase
ROS: Reactive oxygen species
VEGF: Vascular endothelial growth factor
NDUFA4LS: NADH dehydrogenase (ubiquinone) 1 alpha subcomplex, 4-like 2
GPR81: G protein-coupled receptor 81
AMPK: Adenosine-monophosphate activated protein kinase
IκB: Inhibitor of nuclear factor kappa B
TNF-α: Tumor necrosis factor α
ECs: Endothelial cells
Kla: Lysine lactylation
HMGB1: High mobility frame 1
IFN: Interferon

## References

[1] Netea MG, Balkwill F, Chonchol M, Cominelli F, Donath MY, Giamarellos-Bourboulis EJ (2017). A guiding map for inflammation. Nat Immunol.

[2] Medzhitov R (2008). Origin and physiological roles of inflammation. Nature.

[3] Furman D, Campisi J, Verdin E, Carrera-Bastos P, Targ S, Franceschi C (2019). Chronic inflammation in the etiology of disease across the life span. Nat Med.

[4] Fullerton JN, Gilroy DW (2016). Resolution of inflammation: a new therapeutic frontier. Nat Rev Drug Discov.

[5] Franceschi C, Garagnani P, Vitale G, Capri M, Salvioli S (2017). Inflammaging and 'Garb-aging'. Trends Endocrinol Metab.

[6] Shen-Orr SS, Furman D, Kidd BA, Hadad F, Lovelace P, Huang YW (2016). Defective Signaling in the JAK-STAT Pathway Tracks with Chronic Inflammation and Cardiovascular Risk in Aging Humans. Cell Syst.

[7] GBD 2017 Causes of Death Collaborators (2018). Global, regional, and national age-sex-specific mortality for 282 causes of death in 195 countries and territories, 1980–2017: a systematic analysis for the Global Burden of Disease Study 2017. Lancet.

[8] Xu H, Turnquist HR, Hoffman R, Billiar TR (2017). Role of the IL-33-ST2 axis in sepsis. Mil Med Res.

[9] Rudd KE, Johnson SC, Agesa KM, Shackelford KA, Tsoi D, Kievlan DR (2020). Global, regional, and national sepsis incidence and mortality, 1990–2017: analysis for the Global Burden of Disease Study. Lancet.

[10] Dinarello CA (2010). Anti-inflammatory Agents: Present and Future. Cell.

[11] Mita M, Ito M, Harada K, Sugawara I, Ueda H, Tsuboi T, Kitaguchi T (2019). Green Fluorescent Protein-Based Glucose Indicators Report Glucose Dynamics in Living Cells. Anal Chem.

[12] Rabinowitz JD, Enerback S (2020). Lactate: the ugly duckling of energy metabolism. Nat Metab.

[13] Mookerjee SA, Gerencser AA, Nicholls DG, Brand MD (2017). Quantifying intracellular rates of glycolytic and oxidative ATP production and consumption using extracellular flux measurements. J Biol Chem.

[14] Galvan-Pena S, O'Neill LA (2014). Metabolic reprograming in macrophage polarization. Front Immunol.

[15] Kaymak I, Luda KM, Duimstra LR, Ma EH, Longo J, Dahabieh MS (2022). Carbon source availability drives nutrient utilization in CD8(+) T cells. Cell Metab.

[16] Quinn WJ, Jiao J, TeSlaa T, Stadanlick J, Wang Z, Wang L (2020). Lactate Limits T Cell Proliferation via the NAD(H) Redox State. Cell Rep.

[17] Angelin A, Gil-de-Gomez L, Dahiya S, Jiao J, Guo L, Levine MH (2017). Foxp3 Reprograms T Cell Metabolism to Function in Low-Glucose, High-Lactate Environments. Cell Metab.

[18] Watson MJ, Vignali PDA, Mullett SJ, Overacre-Delgoffe AE, Peralta RM, Grebinoski S (2021). Metabolic support of tumour-infiltrating regulatory T cells by lactic acid. Nature.

[19] Zhang D, Tang Z, Huang H, Zhou G, Cui C, Weng Y (2019). Metabolic regulation of gene expression by histone lactylation. Nature.

[20] Warburg O (1925). Iron, the Oxygen-Carrier of Respiration-Ferment. Science.

[21] Krawczyk CM, Holowka T, Sun J, Blagih J, Amiel E, DeBerardinis RJ (2010). Toll-like receptor-induced changes in glycolytic metabolism regulate dendritic cell activation. Blood.

[22] West AP, Brodsky IE, Rahner C, Woo DK, Erdjument-Bromage H, Tempst P (2011). TLR signalling augments macrophage bactericidal activity through mitochondrial ROS. Nature.

[23] Rathinam VAK, Chan FK (2018). Inflammasome, Inflammation, and Tissue Homeostasis. Trends Mol Med.

[24] Palsson-McDermott EM, O'Neill LA (2013). The Warburg effect then and now: from cancer to inflammatory diseases. BioEssays.

[25] Chen Z, Liu M, Li L, Chen L (2018). Involvement of the Warburg effect in non-tumor diseases processes. J Cell Physiol.

[26] Faubert B, Li KY, Cai L, Hensley CT, Kim J, Zacharias LG (2017). Lactate Metabolism in Human Lung Tumors. Cell.

[27] Noe JT, Rendon BE, Geller AE, Conroy LR, Morrissey SM, Young LEA (2021). Lactate supports a metabolic-epigenetic link in macrophage polarization. Sci Adv.

[28] Infantino V, Convertini P, Cucci L, Panaro MA, Di Noia MA, Calvello R (2011). The mitochondrial citrate carrier: a new player in inflammation. Biochem J.

[29] Ivashkiv LB (2020). The hypoxia-lactate axis tempers inflammation. Nat Rev Immunol.

[30] Menkin V, Warner CR (1937). Studies on Inflammation: XIII. Carbohydrate Metabolism, Local Acidosis, and the Cytological Picture in Inflammation. Am J Pathol.

[31] Dubos RJ (1955). The micro-environment of inflammation or Metchnikoff revisited. Lancet.

[32] Edlow DW, Sheldon WH (1971). The pH of inflammatory exudates. Proc Soc Exp Biol Med.

[33] Pucino v, Certo M, Bulusu V, Cucchi D, Goldmann K, Pontarini E (2019). Lactate Buildup at the Site of Chronic Inflammation Promotes Disease by Inducing CD4(+) T Cell Metabolic Rewiring. Cell Metab.

[34] Chen L, Huang L, Gu Y, Cang W, Sun P, Xiang Y. Lactate-lactylation hands between metabolic reprogramming and immunosuppression*.* Int J Mol Sci. 2022;23(19):11943. 10.3390/ijms231911943 PMC9569569 36233246

[35] Srinivas SR, Gopal E, Zhuang L, Itagaki S, Martin PM, Fei YJ (2005). Cloning and functional identification of slc5a12 as a sodium-coupled low-affinity transporter for monocarboxylates (SMCT2). Biochemical Journal.

[36] Liu Z, Meng Z, Li Y, Zhao J, Wu S, Gou S, Wu H (2019). Prognostic accuracy of the serum lactate level, the SOFA score and the qSOFA score for mortality among adults with Sepsis. Scand J Trauma Resusc Emerg Med.

[37] Yang K, Fan M, Wang X, Xu J, Wang Y, Tu F (2022). Lactate promotes macrophage HMGB1 lactylation, acetylation, and exosomal release in polymicrobial sepsis. Cell Death Differ.

[38] Sun L, Suo C, Li ST, Zhang H, Gao P (2018). Metabolic reprogramming for cancer cells and their microenvironment: Beyond the Warburg Effect. Biochim Biophys Acta Rev Cancer.

[39] Corbet C, Pinto A, Martherus R, de Jesus Santiago JP, Polet F, Feron O (2016). Acidosis Drives the Reprogramming of Fatty Acid Metabolism in Cancer Cells through Changes in Mitochondrial and Histone Acetylation. Cell Metab.

[40] Liu PS, Chen YT, Li X, Hsueh PC, Tzeng SF, Chen H, et al. CD40 signal rewires fatty acid and glutamine metabolism for stimulating macrophage anti-tumorigenic functions. Nat Immunol. 2023;24(3):452–62. 10.1038/s41590-023-01430-3 PMC9977680 36823405

[41] Wilson JE (2003). Isozymes of mammalian hexokinase: structure, subcellular localization and metabolic function. J Exp Biol.

[42] Ciscato F, Ferrone L, Masgras I, Laquatra C, Rasola A. Hexokinase 2 in Cancer: A prima donna playing multiple characters*.* Int J Mol Sci. 2021;22(9):4716. 10.3390/ijms22094716 PMC8125560 33946854

[43] Mathupala SP, Ko YH, Pedersen PL (2006). Hexokinase II: cancer's double-edged sword acting as both facilitator and gatekeeper of malignancy when bound to mitochondria. Oncogene.

[44] Shi T, Ma Y, Cao L, Zhan S, Xu Y, Fu F (2019). B7–H3 promotes aerobic glycolysis and chemoresistance in colorectal cancer cells by regulating HK2. Cell Death Dis.

[45] Everts B, Amiel E, Huang SC, Smith AM, Chang CH, Lam WY, et al. TLR-driven early glycolytic reprogramming via the kinases TBK1-IKKvarepsilon supports the anabolic demands of dendritic cell activation. Nat Immunol. 2014;15(4):323–32. 10.1038/ni.2833 PMC4358322 24562310

[46] Vizuete AFK, Froes F, Seady M, Zanotto C, Bobermin LD, Roginski AC (2022). Early effects of LPS-induced neuroinflammation on the rat hippocampal glycolytic pathway. J Neuroinflammation.

[47] Yin Y, Choi SC, Xu Z, Zeumer L, Kanda N, Croker BP, Morel L (2016). Glucose Oxidation Is Critical for CD4+ T Cell Activation in a Mouse Model of Systemic Lupus Erythematosus. J Immunol.

[48] Yin Y, Choi SC, Xu Z, Perry DJ, Seay H, Croker BP (2015). Normalization of CD4+ T cell metabolism reverses lupus. Sci Transl Med.

[49] Hu Y, Cao K, Wang F, Wu W, Mai W, Qiu L (2022). Dual roles of hexokinase 2 in shaping microglial function by gating glycolytic flux and mitochondrial activity. Nat Metab.

[50] Leite TC, Coelho RG, Da Silva D, Coelho WS, Marinho-Carvalho MM, Sola-Penna M (2011). Lactate downregulates the glycolytic enzymes hexokinase and phosphofructokinase in diverse tissues from mice. FEBS Lett.

[51] Ozcan SC, Sarioglu A, Altunok TH, Akkoc A, Guzel S, Guler S (2020). PFKFB2 regulates glycolysis and proliferation in pancreatic cancer cells. Mol Cell Biochem.

[52] Zuo J, Tang J, Lu M, Zhou Z, Li Y, Tian H (2021). Glycolysis Rate-Limiting Enzymes: Novel Potential Regulators of Rheumatoid Arthritis Pathogenesis. Front Immunol.

[53] Houddane A, Bultot L, Novellasdemunt L, Johanns M, Gueuning MA, Vertommen D (2017). Role of Akt/PKB and PFKFB isoenzymes in the control of glycolysis, cell proliferation and protein synthesis in mitogen-stimulated thymocytes. Cell Signal.

[54] Ros S, Schulze A (2013). Balancing glycolytic flux: the role of 6-phosphofructo-2-kinase/fructose 2,6-bisphosphatases in cancer metabolism. Cancer Metab.

[55] Shi L, Pan H, Liu Z, Xie J, Han W (2017). Roles of PFKFB3 in cancer. Signal Transduct Target Ther.

[56] Wong KKL, Liao JZ, Verheyen EM. A positive feedback loop between Myc and aerobic glycolysis sustains tumor growth in a Drosophila tumor model. Elife. 2019;8:e46315. 10.7554/eLife.46315 PMC6636907 31259690

[57] Zhang R, Li R, Liu Y, Li L, Tang Y (2019). The Glycolytic Enzyme PFKFB3 Controls TNF-alpha-Induced Endothelial Proinflammatory Responses. Inflammation.

[58] Schilperoort M, Ngai D, Katerelos M, Power DA, Tabas I (2023). PFKFB2-mediated glycolysis promotes lactate-driven continual efferocytosis by macrophages. Nat Metab.

[59] Jones BC, Pohlmann PR, Clarke R, Sengupta S (2022). Treatment against glucose-dependent cancers through metabolic PFKFB3 targeting of glycolytic flux. Cancer Metastasis Rev.

[60] Gupta V, Bamezai RN (2010). Human pyruvate kinase M2: a multifunctional protein. Protein Sci.

[61] Zhang Z, Deng X, Liu Y, Liu Y, Sun L, Chen F (2019). PKM2, function and expression and regulation. Cell Biosci.

[62] Yang L, Xie M, Yang M, Yu Y, Zhu S, Hou W (2014). PKM2 regulates the Warburg effect and promotes HMGB1 release in sepsis. Nat Commun.

[63] Xie M, Yu Y, Kang R, Zhu S, Yang L, Zeng L (2016). PKM2-dependent glycolysis promotes NLRP3 and AIM2 inflammasome activation. Nat Commun.

[64] Wang J, Yang P, Yu T, Gao M, Liu D, Zhang J (2022). Lactylation of PKM2 Suppresses Inflammatory Metabolic Adaptation in Pro-inflammatory Macrophages. Int J Biol Sci.

[65] Brown TP, Ganapathy V (2020). Lactate/GPR81 signaling and proton motive force in cancer: Role in angiogenesis, immune escape, nutrition, and Warburg phenomenon. Pharmacol Ther.

[66] Pasti AP, Rossi V, Di Stefano G, Brigotti M, Hochkoeppler A. Human lactate dehydrogenase A undergoes allosteric transitions under pH conditions inducing the dissociation of the tetrameric enzyme*.* Biosci Rep. 2022;42(1):BSR20212654. 10.1042/BSR20212654 PMC8799922 35048959

[67] Doherty JR, Cleveland JL (2013). Targeting lactate metabolism for cancer therapeutics. J Clin Invest.

[68] Certo M, Llibre A, Lee W, Mauro C (2022). Understanding lactate sensing and signalling. Trends Endocrinol Metab.

[69] Halestrap AP (2012). The monocarboxylate transporter family–Structure and functional characterization. IUBMB Life.

[70] Felmlee MA, Jones RS, Rodriguez-Cruz V, Follman KE, Morris ME (2020). Monocarboxylate Transporters (SLC16): Function, Regulation, and Role in Health and Disease. Pharmacol Rev.

[71] Braga M, Kaliszczak M, Carroll L, Schug ZT, Heinzmann K, Baxan N, et al. Tracing nutrient flux following monocarboxylate transporter-1 Inhibition with AZD3965*.* Cancers (Basel). 2020;12(6):1703. 10.3390/cancers12061703 PMC7352845 32604836

[72] Sriram R, Van Criekinge M, Hansen A, Wang ZJ, Vigneron DB, Wilson DM (2015). Real-time measurement of hyperpolarized lactate production and efflux as a biomarker of tumor aggressiveness in an MR compatible 3D cell culture bioreactor. NMR Biomed.

[73] Brooks GA (2018). The Science and Translation of Lactate Shuttle Theory. Cell Metab.

[74] Halestrap AP (2013). The SLC16 gene family - structure, role and regulation in health and disease. Mol Aspects Med.

[75] Kong L, Wang Z, Liang X, Wang Y, Gao L, Ma C (2019). Monocarboxylate transporter 1 promotes classical microglial activation and pro-inflammatory effect via 6-phosphofructo-2-kinase/fructose-2, 6-biphosphatase 3. J Neuroinflammation.

[76] Zhang S, Xu W, Wang H, Cao M, Li M, Zhao J (2019). Inhibition of CREB-mediated ZO-1 and activation of NF-kappaB-induced IL-6 by colonic epithelial MCT4 destroys intestinal barrier function. Cell Prolif.

[77] Srinivas SR, Gopal E, Zhuang L, Itagaki S, Martin PM, Fei YJ (2005). Cloning and functional identification of slc5a12 as a sodium-coupled low-affinity transporter for monocarboxylates (SMCT2). Biochem J.

[78] Felmlee MA, Morse BL, Morris ME (2021). gamma-Hydroxybutyric Acid: Pharmacokinetics, Pharmacodynamics, and Toxicology. AAPS J.

[79] Otsuka Y, Furihata T, Nakagawa K, Ohno Y, Reien Y, Ouchi M (2019). Sodium-coupled monocarboxylate transporter 1 interacts with the RING finger- and PDZ domain-containing protein PDZRN3. J Physiol Sci.

[80] Certo M, Tsai CH, Pucino V, Ho PC, Mauro C (2021). Lactate modulation of immune responses in inflammatory versus tumour microenvironments. Nat Rev Immunol.

[81] Proenca-Modena JL, Sesti-Costa R, Pinto AK, Richner JM, Lazear HM, Lucas T (2015). Oropouche virus infection and pathogenesis are restricted by MAVS, IRF-3, IRF-7, and type I interferon signaling pathways in nonmyeloid cells. J Virol.

[82] Wu H, Huang H, Zhao Y (2023). Interplay between metabolic reprogramming and post-translational modifications: from glycolysis to lactylation. Front Immunol.

[83] Zhang W, Wang G, Xu ZG, Tu H, Dai J (2019). Lactate Is a Natural Suppressor of RLR Signaling by Targeting MAVS. Cell.

[84] Korbecki J, Simińska D, Gąssowska-Dobrowolska M, Listos J, Gutowska I, Chlubek D, et al. Chronic and cycling hypoxia: drivers of cancer chronic inflammation through HIF-1 and NF-kappaB activation: A review of the molecular mechanisms*.* Int J Mol Sci. 2021;22(19):10701. 10.3390/ijms221910701 PMC8509318 34639040

[85] Nizet V, Johnson RS (2009). Interdependence of hypoxic and innate immune responses. Nat Rev Immunol.

[86] Firth JD, Ebert BL, Ratcliffe PJ (1995). Hypoxic regulation of lactate dehydrogenase A. Interaction between hypoxia-inducible factor 1 and cAMP response elements. J Biol Chem.

[87] Kim JW, Tchernyshyov I, Semenza GL, Dang CV (2006). HIF-1-mediated expression of pyruvate dehydrogenase kinase: a metabolic switch required for cellular adaptation to hypoxia. Cell Metab.

[88] Min J, Zeng T, Roux M, Lazar D, Chen L, Tudzarova S (2021). The Role of HIF1alpha-PFKFB3 Pathway in Diabetic Retinopathy. J Clin Endocrinol Metab.

[89] Xu Y, An X, Guo X, Habtetsion TG, Wang Y, Xu X (2014). Endothelial PFKFB3 plays a critical role in angiogenesis. Arterioscler Thromb Vasc Biol.

[90] Vegran F, Boidot R, Michiels C, Sonveaux P, Feron O (2011). Lactate influx through the endothelial cell monocarboxylate transporter MCT1 supports an NF-kappaB/IL-8 pathway that drives tumor angiogenesis. Cancer Res.

[91] Sanmarco LM, Rone JM, Polonio CM, Fernandez Lahore G, Giovannoni F, Ferrara K (2023). Lactate limits CNS autoimmunity by stabilizing HIF-1alpha in dendritic cells. Nature.

[92] Morland C, Lauritzen KH, Puchades M, Holm-Hansen S, Andersson K, Gjedde A (2015). The lactate receptor, G-protein-coupled receptor 81/hydroxycarboxylic acid receptor 1: Expression and action in brain. J Neurosci Res.

[93] Ahmed K, Tunaru S, Tang C, Muller M, Gille A, Sassmann A (2010). An autocrine lactate loop mediates insulin-dependent inhibition of lipolysis through GPR81. Cell Metab.

[94] Errea A, Cayet D, Marchetti P, Tang C, Kluza J, Offermanns S (2016). Lactate Inhibits the Pro-Inflammatory Response and Metabolic Reprogramming in Murine Macrophages in a GPR81-Independent Manner. PLoS ONE.

[95] Wang W, Xiao ZD, Li X, Aziz KE, Gan B, Johnson RL, Chen J (2015). AMPK modulates Hippo pathway activity to regulate energy homeostasis. Nat Cell Biol.

[96] Yang K, Xu J, Fan M, Tu F, Wang X, Ha T (2020). Lactate Suppresses Macrophage Pro-Inflammatory Response to LPS Stimulation by Inhibition of YAP and NF-kappaB Activation via GPR81-Mediated Signaling. Front Immunol.

[97] Hoque R, Farooq A, Ghani A, Gorelick F, Mehal WZ (2014). Lactate reduces liver and pancreatic injury in Toll-like receptor- and inflammasome-mediated inflammation via GPR81-mediated suppression of innate immunity. Gastroenterology.

[98] Harun-Or-Rashid M, Inman DM (2018). Reduced AMPK activation and increased HCAR activation drive anti-inflammatory response and neuroprotection in glaucoma. J Neuroinflammation.

[99] Yu H, Lin L, Zhang Z, Zhang H, Hu H (2020). Targeting NF-kappaB pathway for the therapy of diseases: mechanism and clinical study. Signal Transduct Target Ther.

[100] Garcia MA, Gallego P, Campagna M, Gonzalez-Santamaria J, Martinez G, Marcos-Villar L (2009). Activation of NF-kB pathway by virus infection requires Rb expression. PLoS ONE.

[101] Soto-Heredero G, de Las Heras Gomez MM, Gabande-Rodriguez E, Oller J, Mittelbrunn M (2020). Glycolysis - a key player in the inflammatory response. FEBS J.

[102] Bogdan C (2001). Nitric oxide and the immune response. Nat Immunol.

[103] De Bock K, Georgiadou M, Schoors S, Kuchnio A, Wong BW, Cantelmo AR (2013). Role of PFKFB3-driven glycolysis in vessel sprouting. Cell.

[104] Lee HW, Xu Y, Zhu X, Jang C, Choi W, Bae H (2022). Endothelium-derived lactate is required for pericyte function and blood-brain barrier maintenance. EMBO J.

[105] Wu D, Huang RT, Hamanaka RB, Krause M, Oh MJ, Kuo CH, et al. HIF-1α is required for disturbed flow-induced metabolic reprogramming in human and porcine vascular endothelium*.* Elife. 2017;6:e25217. 10.7554/eLife.25217 PMC5495571 28556776

[106] Rossi MT, Langston JC, Singh N, Merali C, Yang Q, Merali S, et al. Molecular Framework of Mouse Endothelial Cell Dysfunction during Inflammation: A Proteomics Approach. Int J Mol Sci. 2022;23(15):8399. 10.3390/ijms23158399 PMC9369400 35955534

[107] Ince C, Mayeux PR, Nguyen T, Gomez H, Kellum JA, Ospina-Tascón GA, et al. The Endothelium in Sepsis. Shock. 2016;45(3):259–70. 10.1097/SHK.0000000000000473 PMC5281063 26871664

[108] Sadiku P, Willson JA, Ryan EM, Sammut D, Coelho P, Watts ER (2021). Neutrophils Fuel Effective Immune Responses through Gluconeogenesis and Glycogenesis. Cell Metab.

[109] Rosales C (2020). Neutrophils at the crossroads of innate and adaptive immunity. J Leukoc Biol.

[110] Borregaard N, Herlin T (1982). Energy metabolism of human neutrophils during phagocytosis. J Clin Invest.

[111] Awasthi D, Nagarkoti S, Sadaf S, Chandra T, Kumar S, Dikshit M (2019). Glycolysis dependent lactate formation in neutrophils: A metabolic link between NOX-dependent and independent NETosis. Biochim Biophys Acta Mol Basis Dis.

[112] Rodriguez-Espinosa O, Rojas-Espinosa O, Moreno-Altamirano MM, Lopez-Villegas EO, Sanchez-Garcia FJ (2015). Metabolic requirements for neutrophil extracellular traps formation. Immunology.

[113] Khatib-Massalha E, Bhattacharya S, Massalha H, Biram A, Golan K, Kollet O (2020). Lactate released by inflammatory bone marrow neutrophils induces their mobilization via endothelial GPR81 signaling. Nat Commun.

[114] Mehla K, Singh PK (2019). Metabolic Regulation of Macrophage Polarization in Cancer. Trends Cancer.

[115] Munder M, Eichmann K, Moran JM, Centeno F, Soler G, Modolell M (1999). Th1/Th2-regulated expression of arginase isoforms in murine macrophages and dendritic cells. J Immunol.

[116] Nareika A, He L, Game BA, Slate EH, Sanders JJ, London SD (2005). Sodium lactate increases LPS-stimulated MMP and cytokine expression in U937 histiocytes by enhancing AP-1 and NF-kappaB transcriptional activities. Am J Physiol Endocrinol Metab.

[117] Kumar A, Pyaram K, Yarosz EL, Hong H, Lyssiotis CA, Giri S, Chang CH (2019). Enhanced oxidative phosphorylation in NKT cells is essential for their survival and function. Proc Natl Acad Sci U S A.

[118] Chang CH, Curtis JD, Maggi LB Jr, Faubert B, Villarino AV, O'Sullivan D, et al. Posttranscriptional control of T cell effector function by aerobic glycolysis. Cell. 2013;153(6):1239–51. 10.1016/j.cell.2013.05.016 PMC3804311 23746840

[119] Wik JA, Skalhegg BS (2022). T Cell Metabolism in Infection. Front Immunol.

[120] Cao J, Liao S, Zeng F, Liao Q, Luo G, Zhou Y (2023). Effects of altered glycolysis levels on CD8(+) T cell activation and function. Cell Death Dis.

[121] Gubser PM, Bantug GR, Razik L, Fischer M, Dimeloe S, Hoenger G (2013). Rapid effector function of memory CD8+ T cells requires an immediate-early glycolytic switch. Nat Immunol.

[122] Ferreira BL, Sousa MB, Leite GGF, Brunialti MKC, Nishiduka ES, Tashima AK (2022). Glucose metabolism is upregulated in the mononuclear cell proteome during sepsis and supports endotoxin-tolerant cell function. Front Immunol.

[123] Tang S, Wan M, Huang W, Stanton RC, Xu Y (2018). Maresins: Specialized Proresolving Lipid Mediators and Their Potential Role in Inflammatory-Related Diseases. Mediators Inflamm.

[124] Kraut JA, Madias NE (2014). Lactic acidosis. N Engl J Med.

[125] Singer M, Deutschman CS, Seymour CW, Shankar-Hari M, Annane D, Bauer M (2016). The Third International Consensus Definitions for Sepsis and Septic Shock (Sepsis-3). JAMA.

[126] Hernandez G, Bellomo R, Bakker J (2019). The ten pitfalls of lactate clearance in sepsis. Intensive Care Med.

[127] Caslin HL, Taruselli MT, Haque T, Pondicherry N, Baldwin EA, Barnstein BO, Ryan JJ (2018). Inhibiting Glycolysis and ATP Production Attenuates IL-33-Mediated Mast Cell Function and Peritonitis. Front Immunol.

[128] Ratter JM, Rooijackers HMM, Hooiveld GJ, Hijmans AGM, de Galan BE, Tack CJ, Stienstra R (2018). In vitro and in vivo Effects of Lactate on Metabolism and Cytokine Production of Human Primary PBMCs and Monocytes. Front Immunol.

[129] Haas R, Smith J, Rocher-Ros V, Nadkarni S, Montero-Melendez T, D'Acquisto F (2015). Lactate Regulates Metabolic and Pro-inflammatory Circuits in Control of T Cell Migration and Effector Functions. PLoS Biol.

[130] Pan RY, He L, Zhang J, Liu X, Liao Y, Gao J (2022). Positive feedback regulation of microglial glucose metabolism by histone H4 lysine 12 lactylation in Alzheimer's disease. Cell Metab.

[131] Coussens LM, Werb Z (2002). Inflammation and cancer. Nature.

[132] Denk D, Greten FR (2022). Inflammation: the incubator of the tumor microenvironment. Trends Cancer.

[133] Feng J, Yang H, Zhang Y, Wei H, Zhu Z, Zhu B (2017). Tumor cell-derived lactate induces TAZ-dependent upregulation of PD-L1 through GPR81 in human lung cancer cells. Oncogene.

[134] Jin P, Jiang J, Zhou L, Huang Z, Qin S, Chen HN (2022). Disrupting metformin adaptation of liver cancer cells by targeting the TOMM34/ATP5B axis. EMBO Mol Med.

[135] Sena P, Mancini S, Benincasa M, Mariani F, Palumbo C, Roncucci L. Metformin induces apoptosis and alters cellular responses to oxidative stress in Ht29 colon cancer cells: preliminary findings*.* Int J Mol Sci. 2018;19(5):1478. 10.3390/ijms19051478 PMC5983851 29772687

[136] Chen S, Zhou X, Yang X, Li W, Li S, Hu Z, et al. Dual blockade of lactate/GPR81 and PD-1/PD-L1 pathways enhances the anti-tumor effects of metformin*.* Biomolecules. 2021;11(9):1373. 10.3390/biom11091373 PMC8466555 34572586

[137] Stern R (2008). Hyaluronidases in cancer biology. Semin Cancer Biol.

[138] Hirschhaeuser F, Sattler UG, Mueller-Klieser W (2011). Lactate: a metabolic key player in cancer. Cancer Res.

[139] Xie Y, Hu H, Liu M, Zhou T, Cheng X, Huang W, Cao L (2022). The role and mechanism of histone lactylation in health and diseases. Front Genet.

[140] Roger T, Lugrin J, Le Roy D, Goy G, Mombelli M, Koessler T (2011). Histone deacetylase inhibitors impair innate immune responses to Toll-like receptor agonists and to infection. Blood.

[141] Stearns-Kurosawa DJ, Osuchowski MF, Valentine C, Kurosawa S, Remick DG (2011). The pathogenesis of sepsis. Annu Rev Pathol.

[142] Hotchkiss RS, Monneret G, Payen D (2013). Immunosuppression in sepsis: a novel understanding of the disorder and a new therapeutic approach. Lancet Infect Dis.

[143] Muszynski JA, Nofziger R, Moore-Clingenpeel M, Greathouse K, Anglim L, Steele L (2018). Early Immune Function and Duration of Organ Dysfunction in Critically III Children with Sepsis. Am J Respir Crit Care Med.

[144] Muszynski JA, Nofziger R, Greathouse K, Steele L, Hanson-Huber L, Nateri J, Hall MW (2014). Early adaptive immune suppression in children with septic shock: a prospective observational study. Crit Care.

[145] Pei F, Yao RQ, Ren C, Bahrami S, Billiar TR, Chaudry IH (2022). Expert consensus on the monitoring and treatment of sepsis-induced immunosuppression. Mil Med Res.

[146] Liu YC, Zou XB, Chai YF, Yao YM (2014). Macrophage polarization in inflammatory diseases. Int J Biol Sci.

[147] Plitas G, Rudensky AY (2016). Regulatory T Cells: Differentiation and Function. Cancer Immunol Res.

[148] Caslin HL, Abebayehu D, Pinette JA, Ryan JJ (2021). Lactate Is a Metabolic Mediator That Shapes Immune Cell Fate and Function. Front Physiol.

[149] Li X, Yang Y, Zhang B, Lin X, Fu X, An Y (2022). Lactate metabolism in human health and disease. Signal Transduct Target Ther.

[150] Chu X, Di C, Chang P, Li L, Feng Z, Xiao S (2021). Lactylated Histone H3K18 as a Potential Biomarker for the Diagnosis and Predicting the Severity of Septic Shock. Front Immunol.

[151] Torres LK, Pickkers P, van der Poll T (2022). Sepsis-Induced Immunosuppression. Annu Rev Physiol.

[152] Li H, Xie X, Guo X, Yang G, Cai B, Liu J (2022). Bifidobacterium spp. and their metabolite lactate protect against acute pancreatitis via inhibition of pancreatic and systemic inflammatory responses. Gut Microbes.

[153] Kotas ME, Medzhitov R (2015). Homeostasis, inflammation, and disease susceptibility. Cell.

[154] Kennedy BK, Berger SL, Brunet A, Campisi J, Cuervo AM, Epel ES (2014). Geroscience: linking aging to chronic disease. Cell.

[155] Vernia P, Caprilli R, Latella G, Barbetti F, Magliocca FM, Cittadini M (1988). Fecal lactate and ulcerative colitis. Gastroenterology.

[156] Hove H, Holtug K, Jeppesen PB, Mortensen PB (1995). Butyrate absorption and lactate secretion in ulcerative colitis. Dis Colon Rectum.

[157] Ranganathan P, Shanmugam A, Swafford D, Suryawanshi A, Bhattacharjee P, Hussein MS (2018). GPR81, a Cell-Surface Receptor for Lactate, Regulates Intestinal Homeostasis and Protects Mice from Experimental Colitis. J Immunol.

[158] Iraporda C, Romanin DE, Bengoa AA, Errea AJ, Cayet D, Foligne B (2016). Local Treatment with Lactate Prevents Intestinal Inflammation in the TNBS-Induced Colitis Model. Front Immunol.

[159] Souto-Carneiro MM, Klika KD, Abreu MT, Meyer AP, Saffrich R, Sandhoff R (2020). Effect of Increased Lactate Dehydrogenase A Activity and Aerobic Glycolysis on the Proinflammatory Profile of Autoimmune CD8+ T Cells in Rheumatoid Arthritis. Arthritis Rheumatol.

[160] Garcia-Carbonell R, Divakaruni AS, Lodi A, Vicente-Suarez I, Saha A, Cheroutre H (2016). Critical Role of Glucose Metabolism in Rheumatoid Arthritis Fibroblast-like Synoviocytes. Arthritis Rheumatol.

[161] Yi O, Lin Y, Hu M, Hu S, Su Z, Liao J (2022). Lactate metabolism in rheumatoid arthritis: Pathogenic mechanisms and therapeutic intervention with natural compounds. Phytomedicine.

[162] Bustamante MF, Oliveira PG, Garcia-Carbonell R, Croft AP, Smith JM, Serrano RL (2018). Hexokinase 2 as a novel selective metabolic target for rheumatoid arthritis. Ann Rheum Dis.

[163] Sun BK, Siprashvili Z, Khavari PA (2014). Advances in skin grafting and treatment of cutaneous wounds. Science.

[164] Greenhalgh DG (2019). Management of Burns. N Engl J Med.

[165] Ferraro NM, Dampier W, Weingarten MS, Spiller KL (2017). Deconvolution of heterogeneous wound tissue samples into relative macrophage phenotype composition via models based on gene expression. Integr Biol (Camb).

[166] Zhang SM, Wei CY, Wang Q, Wang L, Lu L, Qi FZ (2021). M2-polarized macrophages mediate wound healing by regulating connective tissue growth factor via AKT, ERK1/2, and STAT3 signaling pathways. Mol Biol Rep.

[167] Raziyeva K, Kim Y, Zharkinbekov Z, Kassymbek K, Jimi S, Saparov A. Immunology of acute and chronic wound healing*.* Biomolecules. 2021;11(5):700. 10.3390/biom11050700 PMC8150999 34066746

[168] Barrientos S, Stojadinovic O, Golinko MS, Brem H, Tomic-Canic M (2008). Growth factors and cytokines in wound healing. Wound Repair Regen.

[169] Haller HL, Sander F, Popp D, Rapp M, Hartmann B, Demircan M, et al. Oxygen, pH, lactate, and metabolism-how old knowledge and new insights might be combined for new wound treatment. Medicina (Kaunas). 2021;57(11):1190. 10.3390/medicina57111190 PMC8617754 34833408

[170] Westby MJ, Norman G, Watson REB, Cullum NA, Dumville JC (2020). Protease activity as a prognostic factor for wound healing in complex wounds. Wound Repair Regen.

[171] Hunt TK, Aslam RS, Beckert S, Wagner S, Ghani QP, Hussain MZ (2007). Aerobically derived lactate stimulates revascularization and tissue repair via redox mechanisms. Antioxid Redox Signal.

[172] Rendl M, Mayer C, Weninger W, Tschachler E (2001). Topically applied lactic acid increases spontaneous secretion of vascular endothelial growth factor by human reconstructed epidermis. Br J Dermatol.

[173] Nischwitz SP, Popp D, Shubitidze D, Luze H, Zrim R, Klemm K (2022). The successful use of polylactide wound dressings for chronic lower leg wounds: A retrospective analysis. Int Wound J.

[174] Guak HS, Al Habyan S, Ma EH, Aldossary H, Al-Masri M, Won SY (2018). Glycolytic metabolism is essential for CCR7 oligomerization and dendritic cell migration. Nat Commun.

[175] Luo W, Hu H, Chang R, Zhong J, Knabel M, O'Meally R (2011). Pyruvate kinase M2 is a PHD3-stimulated coactivator for hypoxia-inducible factor 1. Cell.

[176] Palsson-McDermott EM, Curtis AM, Goel G, Lauterbach MA, Sheedy FJ, Gleeson LE (2015). Pyruvate kinase M2 regulates Hif-1alpha activity and IL-1beta induction and is a critical determinant of the warburg effect in LPS-activated macrophages. Cell Metab.

[177] Angiari S, Runtsch MC, Sutton CE, Palsson-McDermott EM, Kelly B, Rana N (2020). Pharmacological Activation of Pyruvate Kinase M2 Inhibits CD4(+) T Cell Pathogenicity and Suppresses Autoimmunity. Cell Metab.

[178] Seki SM, Posyniak K, McCloud R, Rosen DA, Fernández-Castañeda A, Beiter RM, et al. Modulation of PKM activity affects the differentiation of T(H)17 cells*.* Sci Signal. 2020;13(655):eaay9217. 10.1126/scisignal.aay9217 PMC8040370 33109748

[179] Qu J, Lu D, Guo H, Miao W, Wu G, Zhou M (2016). PFKFB3 modulates glycolytic metabolism and alleviates endoplasmic reticulum stress in human osteoarthritis cartilage. Clin Exp Pharmacol Physiol.

[180] Wang L, Cao Y, Gorshkov B, Zhou Y, Yang Q, Xu J (2019). Ablation of endothelial Pfkfb3 protects mice from acute lung injury in LPS-induced endotoxemia. Pharmacol Res.

[181] Cao Y, Zhang X, Wang L, Yang Q, Ma Q, Xu J (2019). PFKFB3-mediated endothelial glycolysis promotes pulmonary hypertension. Proc Natl Acad Sci U S A.

[182] Gerriets VA, Kishton RJ, Nichols AG, Macintyre AN, Inoue M, Ilkayeva O (2015). Metabolic programming and PDHK1 control CD4+ T cell subsets and inflammation. J Clin Invest.

[183] Chirasani SR, Leukel P, Gottfried E, Hochrein J, Stadler K, Neumann B (2013). Diclofenac inhibits lactate formation and efficiently counteracts local immune suppression in a murine glioma model. Int J Cancer.

[184] Sun Z, Han Y, Song S, Chen T, Han Y, Liu Y (2019). Activation of GPR81 by lactate inhibits oscillatory shear stress-induced endothelial inflammation by activating the expression of KLF2. IUBMB Life.

[185] Madaan A, Nadeau-Vallee M, Rivera JC, Obari D, Hou X, Sierra EM (2017). Lactate produced during labor modulates uterine inflammation via GPR81 (HCA(1)). Am J Obstet Gynecol.

[186] Zarrouk M, Finlay DK, Foretz M, Viollet B, Cantrell DA (2014). Adenosine-mono-phosphate-activated protein kinase-independent effects of metformin in T cells. PLoS ONE.

[187] Huang ZW, Zhang XN, Zhang L, Liu LL, Zhang JW, Sun YX (2023). STAT5 promotes PD-L1 expression by facilitating histone lactylation to drive immunosuppression in acute myeloid leukemia. Signal Transduct Target Ther.

[188] Xie B, Lin J, Chen X, Zhou X, Zhang Y, Fan M (2023). CircXRN2 suppresses tumor progression driven by histone lactylation through activating the Hippo pathway in human bladder cancer. Mol Cancer.

[189] Xiong J, He J, Zhu J, Pan J, Liao W, Ye H (2022). Lactylation-driven METTL3-mediated RNA m(6)A modification promotes immunosuppression of tumor-infiltrating myeloid cells. Mol Cell.

[190] Lv X, Lv Y, Dai X (2023). Lactate, histone lactylation and cancer hallmarks. Expert Rev Mol Med.

[191] Cui H, Xie N, Banerjee S, Ge J, Jiang D, Dey T (2021). Lung Myofibroblasts Promote Macrophage Profibrotic Activity through Lactate-induced Histone Lactylation. Am J Respir Cell Mol Biol.

[192] Mu X, Shi W, Xu Y, Xu C, Zhao T, Geng B (2018). Tumor-derived lactate induces M2 macrophage polarization via the activation of the ERK/STAT3 signaling pathway in breast cancer. Cell Cycle.

[193] Reber AJ, Chirkova T, Kim JH, Cao W, Biber R, Shay DK, Sambhara S (2012). Immunosenescence and Challenges of Vaccination against Influenza in the Aging Population. Aging Dis.

[194] Martin GS, Mannino DM, Moss M (2006). The effect of age on the development and outcome of adult sepsis. Crit Care Med.

